# Feasibility of Core Antimicrobial Stewardship Interventions in Community Hospitals

**DOI:** 10.1001/jamanetworkopen.2019.9369

**Published:** 2019-08-16

**Authors:** Deverick J. Anderson, Shera Watson, Rebekah W. Moehring, Lauren Komarow, Matthew Finnemeyer, Rebekka M. Arias, Jacqueline Huvane, Carol Bova Hill, Nancie Deckard, Daniel J. Sexton

**Affiliations:** 1 Duke Center for Antimicrobial Stewardship and Infection Prevention, Division of Infectious Diseases, Duke University School of Medicine, Durham, North Carolina; 2 Duke Clinical Research Institute, Durham, North Carolina; 3 The Biostatistics Center, The George Washington University, Rockville, Maryland; 4 Department of Biostatistics, Harvard T.H. Chan School of Public Health, Boston, Massachusetts

## Abstract

**Importance:**

The feasibility of core Infectious Diseases Society of America–recommended antimicrobial stewardship interventions in community hospitals is unknown.

**Objective:**

To determine the feasibility and results of implementing 2 core stewardship intervention strategies in community hospitals.

**Design, Setting, and Participants:**

Three-stage, multicenter, prospective nonrandomized clinical trial with crossover design. The setting was 4 community hospitals in North Carolina (median bed size, 305; range, 102-425). Participants were all patients receiving targeted study antibacterial agents or alternative, nonstudy antibacterial agents. The study dates were October 2014 through October 2015. All statistical analyses were completed as of October 2016.

**Interventions:**

Two antimicrobial stewardship strategies targeted vancomycin hydrochloride, piperacillin-tazobactam, and the antipseudomonal carbapenems on formulary at the study hospitals: (1) modified preauthorization (PA), in which the prescriber had to receive pharmacist approval for continued use of the antibiotic after the first dose, and (2) postprescription audit and review (PPR), in which the pharmacist would engage the prescriber about antibiotic appropriateness after 72 hours of therapy. Two hospitals performed modified PA for 6 months, then PPR for 6 months after a 1-month washout. The other 2 hospitals performed the reverse.

**Main Outcomes and Measures:**

The primary outcome was the feasibility of implementing the interventions, determined by (1) approval by hospital administration and committees at each study hospital; (2) completion of pharmacist training; (3) initiation and implementation as determined by number, type, and outcomes of interventions performed; and (4) time required for interventions. Secondary outcomes included antimicrobial use (days of therapy) compared with matched historical periods and length of hospitalization.

**Results:**

A total of 2692 patients (median age, 65 years; interquartile range, 53-76 years) underwent a study intervention; 1413 (52.5%) were female, 1323 (49.1%) were white, and 1047 (38.9%) were African American. Intervention approvals took a median of 95 days (range, 56-119 days); during these discussions, strict PA was deemed not feasible. Instead, the modified PA intervention was used throughout the study. Pharmacists performed 1456 modified PA interventions (median per hospital, 350 [range, 129-628]) and 1236 PPR interventions (median per hospital, 298 [range, 273-366]). Study antimicrobials were determined to be inappropriate 2 times as often during the PPR period (41.0% [435 of 1060] vs 20.4% [253 of 1243]; *P* < .001). Pharmacists recommended dose change more often during the modified PA intervention (15.9% [232 of 1456] vs 9.6% [119 of 1236]; *P* < .001) and de-escalation during PPR (29.1% [360 of 1236] vs 13.0% [190 of 1456]; *P* < .001). The median time dedicated to the stewardship interventions varied by hospital (range of median hours per week, 5-19). Overall antibiotic use decreased during PPR compared with historical controls (mean [SD] days of therapy per 1000 patient-days, 925.2 [109.8] vs 965.3 [109.4]; mean difference, −40.1; 95% CI, −71.7 to −8.6), but not during modified PA (mean [SD] days of therapy per 1000 patient-days, 931.0 [102.0] vs 926.6 [89.7]; mean difference, 4.4; 95% CI, −55.8 to 64.7).

**Conclusions and Relevance:**

Strict PA was not feasible in the study hospitals. In contrast, PPR was a feasible and effective strategy for antimicrobial stewardship in settings with limited resources and expertise.

**Trial Registration:**

ClinicalTrials.gov identifier: NCT02212808

## Key Points

**Question:**

Are 2 core Infectious Diseases Society of America–recommended antimicrobial stewardship strategies, preauthorization (PA) and postprescription audit and review (PPR), feasible in community hospitals?

**Findings:**

Among 2692 patients in this multicenter nonrandomized clinical trial with crossover design, PPR and a modified PA strategy were feasible; strict PA was not feasible. Postprescription audit and review decreased antimicrobial use and identified more inappropriate antimicrobial therapy, led to more direct interactions with clinicians, and resulted in more antimicrobial de-escalation than the modified PA strategy.

**Meaning:**

Postprescription audit and review is a feasible and effective strategy for antimicrobial stewardship in settings with limited resources and expertise.

## Teaser

This multicenter nonrandomized clinical trial with crossover design study determines the feasibility and results of implementing 2 core stewardship intervention strategies, preauthorization and postprescription audit and review, in community hospitals.

## Introduction

Hospitals across the world continue to observe increasing rates of multidrug-resistant pathogens and *Clostrioides difficile* infection, leading to significant morbidity, mortality, and hospital costs.^1,2,3^ In fact, these trends have become so alarming that the World Health Organization has labeled multidrug-resistant pathogens as one of the top 3 threats to modern health care.^4^

An association between antibiotic exposure and acquired drug resistance is well established. Because 30% to 50% of antibiotic use is inappropriate,^1,5,6^ hospital-level quality improvement programs designed to improve antimicrobial use, namely, antimicrobial stewardship (AS) teams, are an essential intervention to curb these concerning trends.^7^ The Infectious Diseases Society of America (IDSA) recommends the following 2 core strategies for AS: (1) antimicrobial restriction/preauthorization (PA) and (2) postprescription audit and review (PPR) with intervention and feedback.^1,8^ While the need for these core AS strategies is clear, it remains unclear whether they can be effectively used in all health care settings. Stewardship recommendations have been largely generated based on studies performed in large tertiary care hospitals.^8^ In contrast, most health care provided in the United States is provided in community hospitals.^9,10^ These hospitals represent a setting of increased need for stewardship because small, nonteaching community hospitals have the highest rate of antibiotic use in the United States.^11^

However, community hospitals typically have limited or no resources and no trained staff dedicated to AS.^12^ Therefore, understanding which of the core strategies is most feasible in this practice setting would assist in appropriate allocation of limited resources. As a result, we performed this 3-stage, multicenter, prospective nonrandomized clinical trial with crossover design to determine the feasibility and results of implementing 2 core stewardship intervention strategies in community hospitals.

## Methods

### Study Design and Participating Hospitals

We designed this 3-stage, multicenter historically controlled prospective nonrandomized clinical trial with crossover design to determine the feasibility and outcomes from 2 core AS interventions in 4 community hospitals. Stage 1 of the study included 12 months of retrospective data collected from study hospitals from October 6, 2013, through October 5, 2014; stage 2 occurred during the 6-month period from October 6, 2014, through April 3, 2015. After a 1-month washout period, stage 3 occurred during the 6-month period from May 4, 2015, through October 30, 2015 (eFigure 1 in Supplement 1). The crossover approach was used to ensure that unmeasured differences between sites (eg, team dynamics, experience, and support) were balanced between study arms. The trial protocol is available in Supplement 2.

Study hospitals were enrolled from the Duke Antimicrobial Stewardship Outreach Network; all 4 community hospitals were located in North Carolina (median bed size, 305; range, 102-425). These 4 hospitals were selected based on their interest in the study, because they had not yet implemented the core strategies being investigated, and since the pharmacists (all PharmDs) at the study hospitals had not undergone prior training in stewardship. The Duke University Health System Institutional Review Board approved this study; the study was also approved by the institutional review boards at 2 study hospitals, while the other 2 hospitals deferred to the Duke University Health System Institutional Review Board. Because the safety of the interventions were not being tested, this study was deemed minimal risk; therefore, we received a waiver of informed consent to collect data from the patients receiving care at the study hospitals. This study followed the Transparent Reporting of Evaluations With Nonrandomized Designs (TREND) reporting guideline.

### Study Interventions

The study was originally designed to evaluate the following 2 AS interventions: (1) strict PA, in which the pharmacist had to give approval before the first dose, and (2) PPR, in which the pharmacist would engage the prescriber about antibiotic appropriateness after 72 hours of therapy. However, strict PA was deemed infeasible by study hospitals at study onset; in general, hospital leadership had strong perceptions that clinicians did not want to have prescriptions restricted. Therefore, PA was altered to a modified PA intervention, in which the first dose of the antibiotic could be given before the review by the stewardship team. During the PPR intervention, the stewardship team reviewed eligible prescriptions approximately 72 hours (between 48 and 96 hours) after order entry.

Modified PA and PPR interventions were targeted to the following 3 specific study antibiotics: vancomycin hydrochloride, piperacillin-tazobactam, and the antipseudomonal carbapenems on formulary at the study hospitals. All adult and pediatric patients admitted to a study hospital who received one of the study drugs were potentially eligible for the pharmacist intervention. During the modified PA intervention, all prescriptions for study antibiotics during weekday study hours (7 am to 6 pm) required approval by a trained pharmacist after the first dose; orders entered after-hours and during weekends were not evaluated. During the PPR intervention, orders eligible for review on the weekend were assessed by a trained pharmacist the following Monday.

Ultimately, all patients who were prescribed study or nonstudy antimicrobial agents (eTable 1 in Supplement 1) had data collected from their medical records and were included in the analysis, even if the pharmacist failed to perform an intervention on the patient. However, patients who received less than 24 hours of surgical prophylaxis were excluded. Intravenous and oral formulations were included; inhaled or topical administrations were excluded.

### Hospital Assignment

The study hospitals were separated into pairs based on bed size. One hospital from each pair was assigned to a 6-month period of modified PA, followed by a 6-month period of PPR. The other 2 hospitals were assigned to PPR, followed by modified PA. A 1-month washout period occurred between intervention arms.

### Pharmacist Role

One or more pharmacists at each site received standardized training by study personnel to address common questions and anticipated arguments and to establish an enhanced knowledge base regarding the targeted antimicrobials. Each designated pharmacist was also trained in conflict management. As part of the training, study personnel provided suggested criteria for appropriate use of each targeted drug (eAppendix 2 and eAppendix 3 in Supplement 1). In addition, study personnel provided pharmacists with specific clinical pathways for urinary tract infections, community-acquired pneumonia, health care–associated pneumonia, bacteremia, and other uses of targeted antimicrobials. These pathways were modeled after assessment tools published by the Centers for Disease Control and Prevention.^13^

Study hospitals determined the best strategy for identifying eligible patients within their standard workflow. Ultimately, hospitals chose similar strategies: eligible patients were identified using lists generated from pharmacy prescription databases. After identifying an eligible patient, the pharmacist determined if the study antibiotic was necessary and/or required modification after review of the patient’s documented symptoms, pertinent clinical data, and the indication for the study antibiotic documented in the medical record. If recommending a change, the pharmacist was instructed to contact the prescriber to discuss the recommendations. Pharmacists continued to have nonstewardship pharmacy responsibilities, but pharmacy leaders allowed for prioritization of study activities. Pharmacists received feedback on number of interventions during bimonthly conference calls with the study team. Time spent performing the 2 stewardship strategies was supported by grant funds during the study. The central study team was not involved in patient-level interventions at study hospitals at any time.

### Procedures

#### Protocol Approvals

Study personnel worked with study hospital personnel to ensure that protocols for the stewardship interventions were approved at each study hospital. Approval processes varied by study hospital but typically included discussions with and approval by pharmacy leadership, pharmacy and therapeutics, and medical executive committees. Study interventions were not started until approvals were received from all study hospitals.

#### Data Collection

Because all study hospitals were members of the Duke Antimicrobial Stewardship Outreach Network, standardized data collection procedures were in place to collect antimicrobial use, demographics, and outcome data for eligible patients (eAppendix 1 and eTable 3 in Supplement 1). Trained pharmacists and prescribers at each study hospital were sent surveys at the end of each 6-month intervention period to characterize perceptions of the 2 stewardship strategies. Surveys were distributed electronically, on paper, and through person-to-person contact. Patient race/ethnicity was abstracted from the electronic health record.

### Outcomes

The primary outcome was the feasibility of implementing the 2 core stewardship interventions. Feasibility was determined by (1) approval by hospital administration and committees at each study hospital; (2) completion of pharmacist training; (3) initiation and implementation as determined by number, type, and outcomes of interventions performed; and (4) time required for interventions.

Our study included predetermined secondary outcomes as well. These secondary outcomes included the following comparisons of the interventions with the baseline period: (1) antimicrobial use, compared with matched historical periods, measured as days of therapy or number of days a patient received each study and nonstudy antibiotic during the hospital admission, and (2) length of hospitalization. For these secondary outcomes, the at-risk group of patients included all patients who received a study antibiotic or a nonstudy antibiotic. Of note, a nationwide shortage of piperacillin-tazobactam occurred during the study, and this combination drug was not available in hospital B during the PA period.

### Statistical Analysis

Primary feasibility outcome results were descriptive. Process outcomes were analyzed at the patient level and not the cluster level due to the small number of clusters (n = 4). Patients could be included more than once (ie, multiple hospitalizations) and could have had multiple interventions during an admission. Because the result of any pharmacist-initiated intervention is a function not only of the intervention itself but also of the pharmacist, there is potential for site variation in response. Therefore, the results are presented by hospital in addition to being pooled for each intervention. Antibiotic use was analyzed at the hospital level. Days of therapy per 1000 patient-days were evaluated for patients who received a study or nonstudy antibiotic. Use during each study arm was compared with matched historical control groups. The historical period was matched to the intervention period by the dates of the previous year for that intervention period at each hospital. Masking was not used. Discrete variables were summarized using proportions and a χ^2^ test. Continuous variables were summarized using the median and interquartile range (IQR) or the mean and SD or 95% CI as appropriate. All analyses were completed using SAS version 9.4 statistical software (SAS Institute Inc) as of October 2016.

All statistical tests were 2-sided. A threshold of *P* < .05 was used to determine statistical significance.

## Results

A total of 2692 patients underwent a study intervention (median age, 65 years; IQR, 53-76 years); 1413 (52.5%) were female, 1323 (49.1%) were white, and 1047 (38.9%) were African American. A total of 310 different clinicians prescribed a targeted antibiotic (median per hospital, 67; range, 39-126).

### Feasibility

Strict PA was deemed not feasible at study hospitals. Postprescription audit and review and the modified PA interventions were approved at all 4 study hospitals. Approvals required discussion with pharmacy leadership, input from hospital administration, and presentation at and approval by hospital pharmacy and therapeutics committees at all study hospitals. Three of 4 hospitals also required presentation at a medical executive committee. These intervention approval processes took a median of 95 days (range, 56-119 days).

One pharmacist per hospital was responsible for implementing the interventions at 3 study hospitals (Table 1). Hospital A initially targeted 1 pharmacist to implement the interventions but ultimately elected to decentralize the approach and use 4 pharmacists during the second intervention phase. Therefore, a total of 7 pharmacists underwent training for the study. Pharmacist training took a mean (SD) of 2.7 (1.7) hours per pharmacist.

**Table 1.** Study Hospital Characteristics and Number of Interventions Performed During Each Study Phase

Variable	Hospital				Total
	A	B	C	D	
**Study Intervention Sequence**					
Stage 2 intervention	PA	PPR	PA	PPR	NA
Stage 3 intervention	PPR	PA	PPR	PA	NA
**Modified Preauthorization Study Period**					
Pharmacists designated for intervention, No.	1	1	1	1	4
Patient admissions, No.	2853	3882	8148	5766	20 649
Patient-days, No.	11 108	15 075	36 038	24 305	86 526
Interventions, No.	129	349	628	350	1456
**Postprescription Audit and Review Study Period**					
Pharmacists designated for intervention, No.	4	1	1	1	7
Patient admissions, No.	2908	3900	7596	5932	20 336
Patient-days, No.	10 627	16 537	32 488	26 147	85 799
Interventions, No.	273	278	319	366	1236
Infectious diseases physician available for consultation	No	Yes, on-site	Yes, 1 d/wk	No	NA
Infectious diseases physician directs stewardship program	NA	No	No	NA	NA

Abbreviations: NA, not applicable; PA, preauthorization study; PPR, postprescription audit and review study.

All pharmacists responded to the survey, while 86 of 166 clinicians (51.8%) responded to the survey. Pharmacists felt more comfortable discussing antibiotic-related issues after training (Table 2) but had mixed feelings that their recommendations improved patient care. Adding stewardship interventions to current workloads represented a burden to pharmacist workflow. In total, 88.1% (74 of 84) of clinicians in our study hospitals who responded to the survey agreed that AS was important for patients; 57.8% (48 of 83) agreed that pharmacist antibiotic recommendations could improve the care of their patients. While clinicians who responded to the survey were skeptical that pharmacist recommendations changed their individual prescribing strategies, 39 of 80 (48.8%) changed therapy based on pharmacist recommendations. Of note, 31.3% (25 of 80) of clinicians chose nonstudy antibiotics to avoid interaction with the stewardship pharmacist at least once during the intervention periods.

**Table 2.** Pharmacist and Clinician Perceptions of Stewardship Interventions Performed During Preauthorization and Postprescription Audit and Review Study Periods^a^

Variable	Modified Preauthorization	Postprescription Audit and Review
**Pharmacists, No. (%)**		
No.	4	7
I felt comfortable discussing antibiotic management with prescribers at my hospital prior to my participation in the trial		
Agree or strongly agree	3 (75.0)	5 (71.4)
I feel comfortable discussing antibiotic management with prescribers at my hospital now (after training for the trial)		
Agree or strongly agree	4 (100)	7 (100)
In the last 6 mo, my recommendations improved outcomes for patients in my hospital		
Neither agree nor disagree	2 (50.0)	3 (42.9)
Agree	2 (50.0)	4 (57.1)
The stewardship intervention was not burdensome on my workflow		
Disagree	1 (25.0)	2 (28.6)
Neither agree nor disagree	1 (25.0)	3 (42.9)
Agree	2 (50.0)	2 (28.6)
**Clinicians, No./Total No. (%)**		
No.	45	41
I believe antimicrobial stewardship is important for patients at our hospital		
Agree or strongly agree	38/44 (86.4)	36/40 (90.0)
Disagree or strongly disagree	6/44 (13.6)	4/40 (10.0)
I believe the pharmacist recommendations for the study antibiotics helped improve the care of my patients		
Agree or strongly agree	26/44 (59.1)	22/39 (56.4)
Disagree or strongly disagree	18/44 (40.9)	17/39 (43.6)
I believe the pharmacist recommendations for the study antibiotics changed my prescribing strategy		
Agree or strongly agree	16/43 (37.2)	16/40 (40.0)
Disagree or strongly disagree	27/43 (62.8)	24/40 (60.0)
I believe the pharmacist recommendations for the study antibiotics were not burdensome on my workflow		
Agree or strongly agree	28/43 (65.1)	20/38 (52.6)
Disagree or strongly disagree	15/43 (34.9)	18/38 (47.4)
In the last 6 mo, how often did you choose nonstudy antibiotics to avoid the pharmacist?		
At least once	11/43 (25.6)	14/37 (37.8)
Never	32/43 (74.4)	23/37 (62.2)
In the last 6 mo, how often did you change therapy based on the pharmacist’s recommendations?		
At least once	20/43 (46.5)	19/37 (51.4)
Never	23/43 (53.5)	18/37 (48.6)

^a^ Respondents answered each question using a 5-point Likert-type scale. For ease of reporting, some categories have been collapsed. Questions were provided at the end of each study period. Some totals do not sum to heading totals because of missing responses.

### Pharmacist Interventions

Trained pharmacists performed modified PA interventions during 1456 admissions (median per hospital, 350 [range, 129-628]) and PPR interventions during 1236 admissions (median per hospital, 298 [range, 273-366]) during the study (Figure). The patients who received these interventions were generally similar (Table 3). In total, 164 clinicians were contacted for a stewardship intervention (median per hospital, 38; range, 21-68) during the study.

**Figure.**
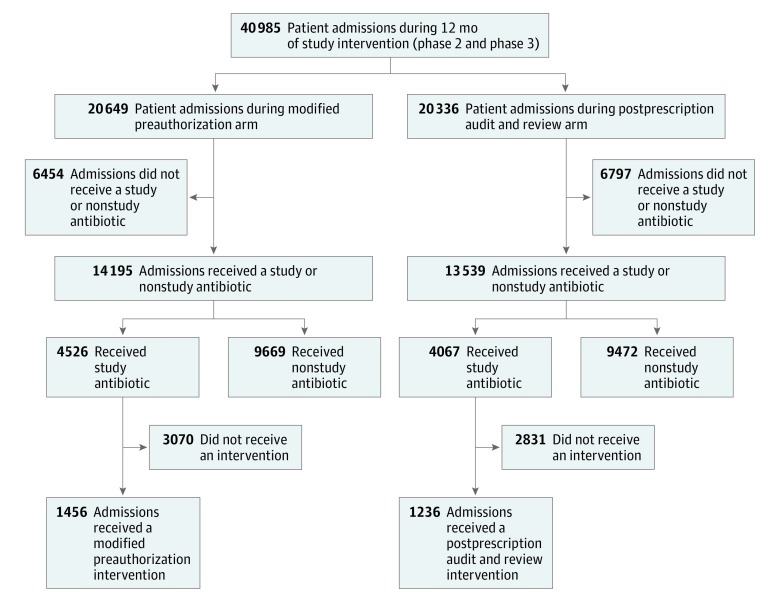
Patient Admissions Shown are patient admissions during the 12 months of active intervention.

**Table 3.** Comparison of Pharmacist Interventions Performed During Modified Preauthorization and Postprescription Audit and Review Study Periods

Variable	Modified Preauthorization (n = 1456)	Postprescription Audit and Review (n = 1236)	*P* Value^a^
**Patient Demographics**			
Age, median (IQR), y	65 (53-75)	66 (54-77)	.21
Female, No. (%)	752 (51.6)	661 (53.5)	.19
Race, No. (%)			
White	696 (47.8)	627 (50.7)	<.001
African American	545 (37.4)	502 (40.6)	
Native American	196 (13.5)	95 (7.7)	
Non-Hispanic ethnicity, No. (%)	1430 (98.2)	1220 (98.7)	.59
**Intervention Descriptors, No./Total No. (%)**			
Most common forms of contact with prescriber			
Phone call	159 (10.9)	383 (31.0)	<.001
In person	155 (10.6)	24 (1.9)	
Medical record review only	843 (57.9)	399 (32.3)	
Intervention on study antibiotic			
Vancomycin	800 (54.9)	700 (56.6)	.36
Piperacillin-tazobactam^b^	482/892 (54.0)	403/775 (52.0)	.40
Carbapenems^b^	77/855 (9.0)	65/813 (8.0)	.82
Antibiotic assessed as not appropriate^c^			
All study antibiotics	253/1243 (20.4)	435/1060 (41.0)	<.001
Vancomycin	83/754 (11.0)	262/707 (37.1)	<.001
Piperacillin-tazobactam	131/522 (25.1)	220/560 (39.3)	<.001
Carbapenems	58/214 (27.1)	38/87 (43.7)	<.001
Antibiotic change recommended	422 (29.0)	479 (38.8)	<.001
De-escalation, any study antibiotic^d^	190 (13.0)	360 (29.1)	<.001
Vancomycin	62/625 (9.9)	218/643 (34.9)	<.001
Piperacillin-tazobactam	108/446 (24.8)	180/497 (36.2)	<.001
Carbapenems	33/208 (15.9)	30/87 (34.5)	<.001
Dose change, any study antibiotic^d^	232 (15.9)	119 (9.6)	<.001
Vancomycin	182/630 (28.9)	91/601 (15.1)	<.001
Piperacillin-tazobactam	84/439 (19.1)	35/511 (6.8)	<.001
Carbapenems	9/235 (3.8)	1/102 (1.0)	.18
Culture data available at the time of review	404 (27.7)	653 (52.8)	<.001
Recommendation followed, if given			
Yes, all	284/443 (64.1)	249/465 (53.5)	.001
Yes, some	67/443 (15.1)	72/465 (15.5)	
No	92/443 (20.8)	144/465 (31.0)	
Infectious diseases consult generated after review	164/1375 (11.9)	91/1138 (8.0)	<.001

Abbreviation: IQR, interquartile range.

^a^ χ^2^ Test for categorical variables and Wilcoxon rank sum test for continuous variables.

^b^ Excludes hospital B, which had a shortage of piperacillin-tazobactam during the study.

^c^ Denominator based on interventions in which appropriateness was documented; denominators for all study antibiotics were 1243 (modified preauthorization) and 1060 (postprescription audit and review).

^d^ De-escalation was defined as transition to a less broad agent or to discontinuing antibiotics. Dose change was defined as a change in the amount or frequency of administration of an antibiotic.

Statistically significant differences were observed across the 2 interventions (Table 3). Pharmacists were more likely to assess appropriateness via medical record review alone during the PA intervention (57.9% [843 of 1456] vs 32.3% [399 of 1236]; *P* < .001) and more likely to call the clinician during the PPR intervention (31.0% [383 of 1236] vs 10.9% [159 of 1456]; *P* < .001). In addition, study antimicrobials were determined to be inappropriate 2 times as often during the PPR period (41.0% [435 of 1060] vs 20.4% [253 of 1243]; *P* < .001). Pharmacists recommended dose change more often during the modified PA intervention (15.9% [232 of 1456] vs 9.6% [119 of 1236]; *P* < .001) and de-escalation during PPR (29.1% [360 of 1236] vs 13.0% [190 of 1456]; *P* < .001).

Intervention and adherence data varied by hospital (eTable 2 in Supplement 1). The median time dedicated to the stewardship interventions varied by hospital (range of median hours per week, 5-19). Overall, pharmacists performed interventions on 2692 of 8593 eligible patients (31.3%). The proportions of pharmacist interventions performed during admissions in which study antibiotics were provided were generally similar during the study periods (Table 4). For example, pharmacists performed interventions during 32.0% (774 of 2419) of admissions in which vancomycin was provided during the PA period and during 29.3% (695 of 2372) of admissions in the PPR period. Pharmacists at hospitals A and D performed a higher proportion of interventions during the PPR period, while pharmacists at hospitals B and C performed a higher proportion of interventions during the PA period.

**Table 4.** Proportions of Pharmacist Interventions Performed During Admissions in Which Study Antibiotics Were Provided

Hospital	Study Antibiotic	Modified Preauthorization		Postprescription Audit and Review	
		Admissions in Which Study Antibiotic Was Administered, No.	Intervention Performed, No. (%)	Admissions in Which Study Antibiotic Was Administered, No.	Intervention Performed, No. (%)
All	Vancomycin	2419	774 (32.0)	2372	695 (29.3)
	Piperacillin-tazobactam	1418	515 (36.3)	1496	570 (38.1)
	Carbapenems	419	208 (49.6)	199	84 (42.2)
A	Vancomycin	382	68 (17.8)	406	175 (43.1)
	Piperacillin-tazobactam	339	76 (22.4)	296	138 (46.6)
	Carbapenems	37	9 (24.3)	44	27 (61.4)
B	Vancomycin	594	269 (45.3)	595	203 (34.1)
	Piperacillin-tazobactam	127	45 (35.4)	475	168 (35.4)
	Carbapenems	241	133 (55.2)	54	21 (38.9)
C	Vancomycin	875	222 (25.4)	816	102 (12.5)
	Piperacillin-tazobactam	499	222 (44.5)	358	82 (22.9)
	Carbapenems	58	22 (37.9)	39	2 (5.1)
D	Vancomycin	568	215 (37.9)	555	215 (38.7)
	Piperacillin-tazobactam	453	172 (38.0)	367	182 (49.6)
	Carbapenems	83	44 (53.0)	62	34 (54.8)

### Patient Outcomes

The results of stewardship interventions on antibiotic use were evaluated by comparing data from patients who received a study or nonstudy antibiotic during each of the intervention periods with a matched historical baseline. Eligible patients in the historical baseline, PA, and PPR periods were similar (eTable 2 in Supplement 1).

Changes in antibiotic use data during each intervention arm are summarized in eFigure 2, eFigure 3, and eTable 4 in Supplement 1. Overall antibiotic use decreased during PPR compared with historical controls (mean [SD] days of therapy per 1000 patient-days, 925.2 [109.8] vs 965.3 [109.4]; mean difference, −40.1; 95% CI, −71.7 to −8.6), but not during modified PA (mean [SD] days of therapy per 1000 patient-days. 931.0 [102.0] vs 926.6 [89.7]; mean difference, 4.4; 95% CI, −55.8 to 64.7). Two of 3 evaluable hospitals had a decrease in use of piperacillin-tazobactam in both PA and PPR arms, but the mean decreases were not statistically significant. In contrast, vancomycin use increased in 3 of 4 hospitals in both PA and PPR arms, but these increases were also not statistically significant. Changes in use varied by antimicrobial agent and by hospital (eTable 3 in Supplement 1).

Length of hospitalization was essentially unchanged throughout the study. The median length of hospitalization per admission was 2 days (IQR, 1-5 days) during the PA intervention period and 3 days (IQR, 1-5 days) during the PPR intervention period. The median length of hospitalization per admission was 3 days (IQR, 1-5 days) during the historical baseline.

## Discussion

Antimicrobial stewardship is a key strategy to combat the emergence of antimicrobial resistance. Developing and implementing stewardship strategies in all health care settings remains a priority. In particular, feasible and effective stewardship strategies are needed in community hospitals, where most health care in the United States is provided.^9,10^ Our study is the first multicenter trial to date to determine and compare the feasibility of initiating and performing 2 of the IDSA’s core stewardship strategies in community hospitals. Strict PA was not feasible in our study hospitals. In contrast, PPR and the modified, “first dose free” PA strategy were feasible. Postprescription audit and review identified more inappropriate antimicrobial therapy, led to more direct interactions with clinicians, and resulted in more antimicrobial de-escalation than the modified PA strategy. Postprescription audit and review led to a decrease in antimicrobial use compared with matched controls.

Almost 90% (74 of 84) of clinicians in our study hospitals who responded to the survey agreed that AS was important for patients; more than 55% (48 of 83) agreed that pharmacist antibiotic recommendations could improve the care of their patients. Buckel et al^14^ polled pharmacists and prescribers at 20 community hospitals; both groups agreed that antimicrobials were overused in their hospitals. This increasing awareness of the importance of stewardship and acceptance of stewardship strategies in the community hospital setting is important. Compared with larger hospitals, small hospitals have similar rates of *C difficile* and drug-resistant bacteria,^15^ are less likely to have core stewardship elements in place,^16^ and have the highest rates of antimicrobial use.^17^ Overall, patients and prescribers in community hospitals need and want the benefit of strong AS programs.

Given that stewardship is so clearly needed in community hospitals, the inevitable question becomes what stewardship interventions should community hospitals prioritize? The Centers for Disease Control and Prevention has published a guidance document for implementation of AS at small and critical access hospitals.^18^ That document provides several options but does not help community hospitals determine which stewardship interventions are most feasible. Numerous studies describing stewardship interventions in community hospitals have been published, including use of rapid diagnostic testing,^19,20^ remote medical record access,^21,22^ antibiotic use guidance and bundles,^23^ decentralized pharmacy interventions,^24^ and review of study antibiotics.^25,26,27,28^ However, the generalizability of the findings from these studies is limited by quasi-experimental design, single-center experiences, requirement of infectious diseases (ID) physician interventions, and differences in resources across hospital and geographic settings.

To our knowledge, this study is the first prospective, multicenter study to determine and compare the feasibility of PA vs PPR in community hospitals. Two other studies compared the effectiveness of these 2 strategies, both performed at large academic tertiary care centers. Mehta et al^29^ performed a quasi-experimental study comparing antimicrobial use as the hospital transitioned from PA to PPR for 3 broad-spectrum antibiotics (cefepime hydrochloride, piperacillin-tazobactam, and vancomycin). Total antimicrobial use, broad-spectrum anti–gram negative antimicrobial use, cefepime use, piperacillin-tazobactam use, and length of hospitalization all significantly increased after initiation of PPR. Tamma et al^30^ performed a prospective crossover trial among 4 medicine teams over a 4-month period. Two teams performed PA and 2 teams performed PPR for 4 months. After a 1-month washout period, teams crossed over to the other strategy. In contrast to the results by Mehta et al,^29^ Tamma et al^30^ found that antimicrobial use decreased and was more frequently guideline adherent in the PPR arm, although inappropriate initial therapy was more common.

The IDSA and the Society for Healthcare Epidemiology of America recommend that ID physicians and ID-trained pharmacists lead stewardship teams.^8^ However, ID physicians are not available or are unwilling to invest time into stewardship in many community hospitals either due to disinterest or lack of reimbursement. Our findings suggest that PPR is a better choice than PA for stewardship teams in community hospitals with limited resources, particularly when stewardship interventions must be completed by a pharmacist. While a recent editorial from stewardship experts recommended PA of designated antibiotics in community hospitals to improve empirical antibiotic therapy,^31^ our data suggest that this strategy is not feasible in all community hospital settings. Hospitals in our study were unwilling to adopt this approach without constant availability of ID-trained clinicians. In contrast, a modified PA approach (ie, first dose free) and PPR were both feasible, and both identified numerous opportunities to improve antimicrobial use. Pharmacists identified more inappropriate antimicrobial use and had more direct interactions with clinicians when performing PPR. Recommendations made during PPR were more likely to lead to de-escalation of therapy, while the most common recommendation during PA was dose adjustment. Likely as a result of these interventions and interactions, use of antibiotics was lower during the PPR intervention compared with historical controls. Even modest decreases in antimicrobial use are valuable, particularly when potentially achievable in the more than 3000 community hospitals in the United States. The above findings mirror the results from a recent cluster randomized trial of stewardship implementation at 15 community hospitals in a single health system, in which use of PPR led to decreases in antimicrobial use.^32^ Of note, the biggest reduction in antimicrobial use in the trial was observed in hospitals that had access both to PPR and to ID-trained clinicians to oversee a strict PA approach.

While more feasible and more effective, PPR in community hospitals can be made more efficient, as pharmacists intervened on less than 40% (2692 of 8593) of qualifying patients during our study. Pharmacists in our study hospitals had multiple responsibilities, including activities unrelated to stewardship. These multiple responsibilities may have led to the belief among participating pharmacists that these stewardship interventions were burdensome. In addition, these interventions were only performed during regular business hours. Community hospitals may benefit from providing dedicated resources (time and personnel) that can perform stewardship interventions at all times and offer sustainability for the program. In addition, rapid diagnostics and electronic health record data review tools can help streamline PPR and other stewardship activities, although many community hospitals do not have these resources in place.

### Limitations

Our study has limitations. First, our study hospitals were community hospitals in North Carolina that received support to participate in the study and were interested in participating; therefore, our results may have limited generalizability. In contrast, our study represents the first multicenter prospective evaluation to date, to our knowledge, of the feasibility of core stewardship interventions in the community hospital setting. Second, our study was designed to test feasibility. Although we identified a significant decrease in overall antibiotic use during the PPR intervention, our study was largely underpowered to evaluate the results of these interventions on antimicrobial use, particularly for individual agents. In addition, our intervention occurred contemporaneously with the onset of the Centers for Medicare & Medicaid Services sepsis core measure (SEP-1, in October 2015); preliminary data from our network suggest that community hospitals increased antibiotic use in anticipation of this measure.^33^ Third, an unexpected shortage of piperacillin-tazobactam occurred during our study. This shortage limited our ability to measure use outcomes in one study hospital. Fourth, conclusions from our survey data are limited by moderate response rates from clinicians and by potential recall bias. Fifth, pharmacists performed intervention and data collection activities, and no second-level reviews were performed to adjudicate the determination of appropriateness; therefore, pharmacist perceptions of burden may have been influenced by the time required for data collection and entry, and appropriateness data may be biased by individual pharmacist’s perceptions.

## Conclusions

Data from our 3-stage, multicenter, historically controlled prospective nonrandomized clinical trial with crossover design add to the growing literature that stewardship can be successfully performed in community hospital settings. Active, core stewardship interventions were feasible in our community hospitals, although true PA or “restriction” of antibiotics was not. These interventions led to more interactions between pharmacists and prescribers, providing additional opportunities to optimize antimicrobial therapy. More specifically, PPR in our study led to more interventions, particularly de-escalation, which likely influenced overall antimicrobial use. Ultimately, for hospitals to be most efficient, stewardship teams in community hospitals will need to have dedicated time and resources to complete stewardship interventions that fit their local environment.

## Notes

Supplement 1. **eAppendix 1.** Supplement to the Methods **eFigure 1.** Schema for the Three-Stage, Multicenter Historically Controlled Crossover Trial Study Design **eFigure 2.** Impact of Core Stewardship Interventions on Days of Therapy (DOT) of Antimicrobials per 1,000 Patient Days **eFigure 3.** Changes in Antibiotic Utilization During the 3-Stage Prospective Crossover Study **eTable 1.** List of All Study and Non-Study Antimicrobial Agents **eTable 2.** Comparison of Historical Baseline vs. Intervention Data—Description of Hospitalizations During Which Patients Received Study or Non-Study Antibiotics **eTable 3.** Patient Demographics, Intervention Descriptors and Compliance Data by Study Hospital **eTable 4.** Impact of Core Stewardship Interventions on Days of Therapy (DOT) of Antimicrobials per 1,000 Patient Days Among All Patients Who Received Study or Non-Study Antimicrobials Compared to Matched Historical Baseline **eAppendix 2.** Guidance for Use of Targeted Antibiotics Targeted Antibiotics—Pre-Authorization **eReferences 1.** **eAppendix 3.** General Guidance for Use of Targeted Antimicrobials—Post-prescription Review **eReferences 2.**

Supplement 2. **Trial Protocol**

## References

[1] Dellit TH, Owens RC, McGowan JE Jr, ; Infectious Diseases Society of America; Society for Healthcare Epidemiology of America Infectious Diseases Society of America and the Society for Healthcare Epidemiology of America guidelines for developing an institutional program to enhance antimicrobial stewardship. Clin Infect Dis. 2007;44(2):-. doi:10.1086/510393 17173212

[2] Miller BA, Chen LF, Sexton DJ, Anderson DJ Comparison of the burdens of hospital-onset, healthcare facility–associated *Clostridium difficile* infection and of healthcare-associated infection due to methicillin-resistant *Staphylococcus aureus* in community hospitals. Infect Control Hosp Epidemiol. 2011;32(4):387-390. doi:10.1086/659156 21460491

[3] Sievert DM, Ricks P, Edwards JR, ; National Healthcare Safety Network (NHSN) Team and Participating NHSN Facilities Antimicrobial-resistant pathogens associated with healthcare-associated infections: summary of data reported to the National Healthcare Safety Network at the Centers for Disease Control and Prevention, 2009-2010. Infect Control Hosp Epidemiol. 2013;34(1):1-14. doi:10.1086/668770 23221186

[4] World Health Organization Antibiotic resistance. http://www.who.int/mediacentre/factsheets/antibiotic-resistance/en/. Published February 5, 2018. Accessed July 1, 2018.

[5] Moehring RW, Anderson DJ Antimicrobial stewardship as part of the infection prevention effort. Curr Infect Dis Rep. 2012;14(6):592-600. doi:10.1007/s11908-012-0289-x 22961224

[6] Cosgrove SE, Seo SK, Bolon MK, ; CDC Prevention Epicenter Program Evaluation of postprescription review and feedback as a method of promoting rational antimicrobial use: a multicenter intervention. Infect Control Hosp Epidemiol. 2012;33(4):374-380. doi:10.1086/664771 22418633

[7] Society for Healthcare Epidemiology of America; Infectious Diseases Society of America; Pediatric Infectious Diseases Society Policy statement on antimicrobial stewardship by the Society for Healthcare Epidemiology of America (SHEA), the Infectious Diseases Society of America (IDSA), and the Pediatric Infectious Diseases Society (PIDS). Infect Control Hosp Epidemiol. 2012;33(4):322-327. doi:10.1086/665010 22418625

[8] Barlam TF, Cosgrove SE, Abbo LM, Implementing an antibiotic stewardship program: guidelines by the Infectious Diseases Society of America and the Society for Healthcare Epidemiology of America. Clin Infect Dis. 2016;62(10):e51-e77. doi:10.1093/cid/ciw118 27080992 PMC5006285

[9] National Center for Health Statistics Health, United States, 2011: with special feature on socioeconomic status and health. http://www.cdc.gov/nchs/data/hus/hus11.pdf. Published 2012. Accessed September 4, 2013. 22812021

[10] Agency for Healthcare Research and Quality Healthcare Cost and Utilization Project: statistics on hospital stays. http://hcupnet.ahrq.gov/. Accessed September 4, 2013.

[11] Baggs J, Fridkin SK, Pollack LA, Srinivasan A, Jernigan JA Estimating national trends in inpatient antibiotic use among US hospitals from 2006 to 2012. JAMA Intern Med. 2016;176(11):1639-1648. doi:10.1001/jamainternmed.2016.5651 27653796 PMC10863902

[12] Ohl CA, Dodds Ashley ES Antimicrobial stewardship programs in community hospitals: the evidence base and case studies. Clin Infect Dis. 2011;53(suppl 1):S23-S28. doi:10.1093/cid/cir365 21795725

[13] Centers for Disease Control and Prevention Antibiotic prescribing and use in hospitals and long-term care: implentation resources. https://www.cdc.gov/antibiotic-use/healthcare/implementation.html. Published 2018. Accessed January 31, 2018.

[14] Buckel WR, Hersh AL, Pavia AT, Jones PS, Owen-Smith AA, Stenehjem E Antimicrobial stewardship knowledge, attitudes, and practices among health care professionals at small community hospitals. Hosp Pharm. 2016;51(2):149-157. doi:10.1310/hpj5102-149 PMC11089601 38746767

[15] Gandra S, Trett A, Klein EY, Laxminarayan R Is antimicrobial resistance a bigger problem in tertiary care hospitals than in small community hospitals in the United States? Clin Infect Dis. 2017;65(5):860-863. doi:10.1093/cid/cix413 28472253

[16] O’Leary EN, van Santen KL, Webb AK, Pollock DA, Edwards JR, Srinivasan A Uptake of antibiotic stewardship programs in US acute care hospitals: findings from the 2015 National Healthcare Safety Network Annual Hospital Survey. Clin Infect Dis. 2017;65(10):1748-1750. doi:10.1093/cid/cix651 29020178

[17] Stenehjem E, Hersh AL, Sheng X, Antibiotic use in small community hospitals. Clin Infect Dis. 2016;63(10):1273-1280. doi:10.1093/cid/ciw588 27694483

[18] Centers for Disease Control and Prevention Antibiotic prescribing and use in hospitals and long-term care: implementation of antibiotic stewardship core elements at small and critical access hospitals. https://www.cdc.gov/antibiotic-use/healthcare/implementation/core-elements-small-critical.html. Accessed January 19, 2019.

[19] Lockwood AM, Perez KK, Musick WL, Integrating rapid diagnostics and antimicrobial stewardship in two community hospitals improved process measures and antibiotic adjustment time. Infect Control Hosp Epidemiol. 2016;37(4):425-432. doi:10.1017/ice.2015.313 26738993

[20] Box MJ, Sullivan EL, Ortwine KN, Outcomes of rapid identification for gram-positive bacteremia in combination with antibiotic stewardship at a community-based hospital system. Pharmacotherapy. 2015;35(3):269-276. doi:10.1002/phar.1557 25809178

[21] Wood ZH, Nicolsen NC, Allen N, Cook PP Remote antimicrobial stewardship in community hospitals. Antibiotics (Basel). 2015;4(4):605-616. doi:10.3390/antibiotics4040605 27025642 PMC4790314

[22] Yam P, Fales D, Jemison J, Gillum M, Bernstein M Implementation of an antimicrobial stewardship program in a rural hospital. Am J Health Syst Pharm. 2012;69(13):1142-1148. doi:10.2146/ajhp110512 22722593

[23] Borde JP, Batin N, Rieg S, Adherence to an antibiotic stewardship bundle targeting *Staphylococcus aureus* blood stream infections at a 200-bed community hospital. Infection. 2014;42(4):713-719. doi:10.1007/s15010-014-0633-1 24889541

[24] Smith T, Philmon CL, Johnson GD, Antimicrobial stewardship in a community hospital: attacking the more difficult problems. Hosp Pharm. 2014;49(9):839-846. doi:10.1310/hpj4909-839 25477615 PMC4252187

[25] Storey DF, Pate PG, Nguyen AT, Chang F Implementation of an antimicrobial stewardship program on the medical-surgical service of a 100-bed community hospital. Antimicrob Resist Infect Control. 2012;1(1):32. doi:10.1186/2047-2994-1-32 23043720 PMC3499185

[26] Day SR, Smith D, Harris K, Cox HL, Mathers AJ An infectious diseases physician–led antimicrobial stewardship program at a small community hospital associated with improved susceptibility patterns and cost-savings after the first year. Open Forum Infect Dis. 2015;2(2):ofv064. doi:10.1093/ofid/ofv064 26110166 PMC4473105

[27] Bartlett JM, Siola PL Implementation and first-year results of an antimicrobial stewardship program at a community hospital. Am J Health Syst Pharm. 2014;71(11):943-949. doi:10.2146/ajhp130602 24830998

[28] Libertin CR, Watson SH, Tillett WL, Peterson JH Dramatic effects of a new antimicrobial stewardship program in a rural community hospital. Am J Infect Control. 2017;45(9):979-982. doi:10.1016/j.ajic.2017.03.024 28526311

[29] Mehta JM, Haynes K, Wileyto EP, ; Centers for Disease Control and Prevention Epicenter Program Comparison of prior authorization and prospective audit with feedback for antimicrobial stewardship. Infect Control Hosp Epidemiol. 2014;35(9):1092-1099. doi:10.1086/677624 25111916 PMC4198070

[30] Tamma PD, Avdic E, Keenan JF, What is the more effective antibiotic stewardship intervention: preprescription authorization or postprescription review with feedback? Clin Infect Dis. 2017;64(5):537-543. 27927861 10.1093/cid/ciw780 PMC6248350

[31] Stenehjem E, Hyun DY, Septimus E, Antibiotic stewardship in small hospitals: barriers and potential solutions. Clin Infect Dis. 2017;65(4):691-696. doi:10.1093/cid/cix407 28472291

[32] Stenehjem E, Hersh AL, Buckel WR, Impact of implementing antibiotic stewardship programs in 15 small hospitals: a cluster-randomized intervention. Clin Infect Dis. 2018;67(4):525-532. doi:10.1093/cid/ciy155 29790913

[33] Anderson DJ, Dodds Ashley E, Parish A, The impact of the CMS SEP-1 Core Measure on antimicrobial utilization: a multicenter interrupted time-series (ITS) analysis. Open Forum Infect Dis. 2018;5(suppl 1):S17. doi:10.1093/ofid/ofy209.039

