# Supplementary material for: Feasibility of Core Antimicrobial Stewardship Interventions in Community Hospitals
Source: JAMA Netw Open. 2019 Aug 16;2(8):e199369. doi: 10.1001/jamanetworkopen.2019.9369 (PMC6704742; doi:10.1001/jamanetworkopen.2019.9369)
Supplement: Supplement 1. — eAppendix 1. Supplement to the Methods eFigure 1. Schema for the Three-Stage, Multicenter Historically Controlled Crossover Trial Study Design eFigure 2. Impact of Core Stewardship Interventions on Days of Therapy (DOT) of Antimicrobials per 1,000 Patient Days eFigure 3. Changes in Antibiotic Utilization During the 3-Stage Prospective Crossover Study eTable 1. List of All Study and Non-Study Antimicrobial Agents eTable 2. Comparison of Historical Baseline vs. Intervention Data—Description of Hospitalizations During Which Patients Received Study or Non-Study Antibiotics eTable 3. Patient Demographics, Intervention Descriptors and Compliance Data by Study Hospital eTable 4. Impact of Core Stewardship Interventions on Days of Therapy (DOT) of Antimicrobials per 1,000 Patient Days Among All Patients Who Received Study or Non-Study Antimicrobials Compared to Matched Historical Baseline eAppendix 2. Guidance for Use of Targeted Antibiotics Targeted Antibiotics—Pre-Authorization eReferences 1. eAppendix 3. General Guidance for Use of Targeted Antimicrobials—Post-prescription Review eReferences 2. [file jamanetwopen-2-e199369-s001.pdf]

Supplementary Online Content

Anderson DJ, Watson S, Moehring RW, et al; Antibacterial Resistance Leadership Group (ARLG). Feasibility of core antimicrobial stewardship interventions in community hospitals. *JAMA Netw Open*. 2019;2(8):e199369. doi:10.1001/jamanetworkopen.2019.9369

eAppendix 1. Supplement to the Methods

eFigure 1. Schema for the Three-Stage, Multicenter Historically Controlled Crossover Trial Study Design

eFigure 2. Impact of Core Stewardship Interventions on Days of Therapy (DOT) of Antimicrobials per 1,000 Patient Days

eFigure 3. Changes in Antibiotic Utilization During the 3-Stage Prospective Crossover Study

eTable 1. List of All Study and Non-Study Antimicrobial Agents

eTable 2. Comparison of Historical Baseline vs. Intervention Data—Description of Hospitalizations During Which Patients Received Study or Non-Study Antibiotics

eTable 3. Patient Demographics, Intervention Descriptors and Compliance Data by Study Hospital

eTable 4. Impact of Core Stewardship Interventions on Days of Therapy (DOT) of Antimicrobials per 1,000 Patient Days Among All Patients Who Received Study or Non-Study Antimicrobials Compared to Matched Historical Baseline

eAppendix 2. Guidance for Use of Targeted Antibiotics Targeted Antibiotics—Pre-Authorization

eReferences 1.

eAppendix 3. General Guidance for Use of Targeted Antimicrobials—Post-prescription Review

eReferences 2.

This supplementary material has been provided by the authors to give readers additional information about their work.

© 2019 Anderson DJ et al. *JAMA Network Open*.

eAppendix 1. Supplement to the Methods

Interventions – Clinical pharmacists determined appropriateness of therapy based on study clinical pathways, their baseline knowledge, and acquired knowledge derived by training from study personnel. Study hospitals determined the best strategy for identifying eligible patients within their standard workflow. Ultimately, hospitals chose similar strategies: eligible patients were identified using lists generated from pharmacy prescription databases.

The trained pharmacists at each study hospital were involved in two critical components of the protocol: a) completing the intervention and b) documenting the outcome of the intervention. All interactions with prescribers were documented in a REDCap study database, including documenting which targeted antibiotic was requested, the prescriber's rationale for requesting the targeted antibiotic, the patient's symptoms and pertinent clinical data at the time of the request, whether the request met the criteria for use of the targeted antibiotic (or not), and the pharmacist's recommendation. If the pharmacist concluded that the antibiotic was appropriate following audit and review, then he or she documented that the antibiotic was appropriate but was not required to contact the prescriber. For the purposes of this study, a "dose change" intervention occurred when the pharmacist recommended a change in the amount or frequency or the antibiotic under review. A "de-escalation" intervention occurred when the pharmacist recommended that a different, less broad antibiotic be used (or that antibiotics be stopped).

For both strategies, the pharmacist reviewed the patient's chart approximately five days after interacting with the prescriber to document whether the recommendation was followed. The pharmacist also documented the recommended duration of antibiotic, whether the dose of the antibiotic was appropriate, and the ultimate infection diagnosis, if known. Pharmacists were instructed

© 2019 Anderson DJ et al. *JAMA Network Open*.

not to consider time collecting data when documenting the amount of time required for the interventions.

Data Collection – As all study hospitals were members of DASON, standardized data collection procedures were in place to collect antimicrobial utilization, demographic, and outcomes data for eligible patients. All study hospitals had electronic systems that tracked antimicrobial prescriptions through electronic medication administration records (eMAR), allowing for data collection for individual doses of medications. Utilization data were collected for targeted antibiotics and alternative antimicrobials, including fluoroquinolones, cephalosporins, and anti-methicillin-resistant *Staphylococcus aureus* (MRSA) systemic antimicrobials (e.g., daptomycin, linezolid, ceftaroline, clindamycin, and trimethoprim-sulfamethoxazole [TMP-SMX]) (See Supplementary Table 1 for a more detailed list). DASON routine practice is to remove Protected Health Information (PHI) from data feeds from member hospitals. However, medical record number (MRN) was included in data transmission for this study in order to allow linking of the above data tables with intervention-specific data entered by pharmacists during the study. MRN was replaced with a non-PHI ID after these tables were linked prior to transfer for statistical analysis.

© 2019 Anderson DJ et al. *JAMA Network Open*.

eFigure 1. Schema for the Three-Stage, Multicenter Historically Controlled Crossover Trial Study Design

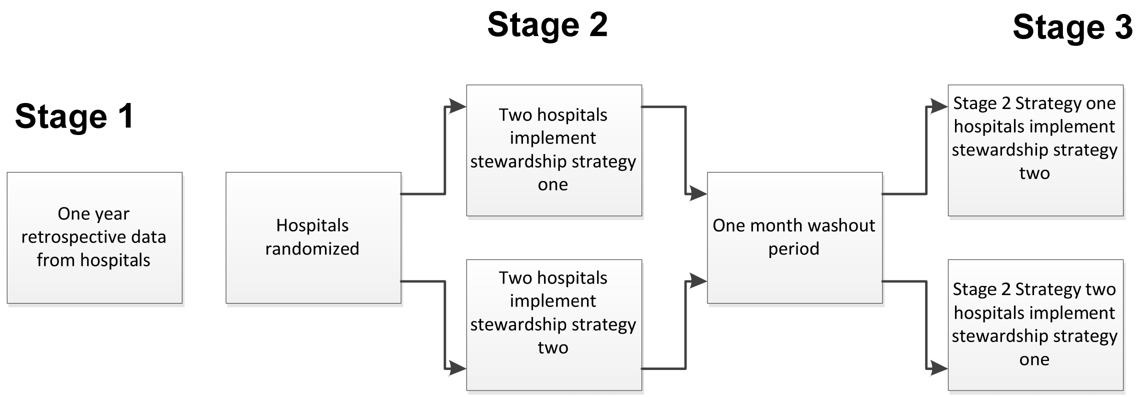

© 2019 Anderson DJ et al. *JAMA Network Open*.

eFigure 2a-e. Impact of Core Stewardship Interventions on Days of Therapy (DOT) of Antimicrobials per 1,000 Patient Days

2a. Utilization of all study and non-study antimicrobials among patients who received any antibiotic during the pre-authorization phase (A) and post-prescription audit and feedback (B) interventions

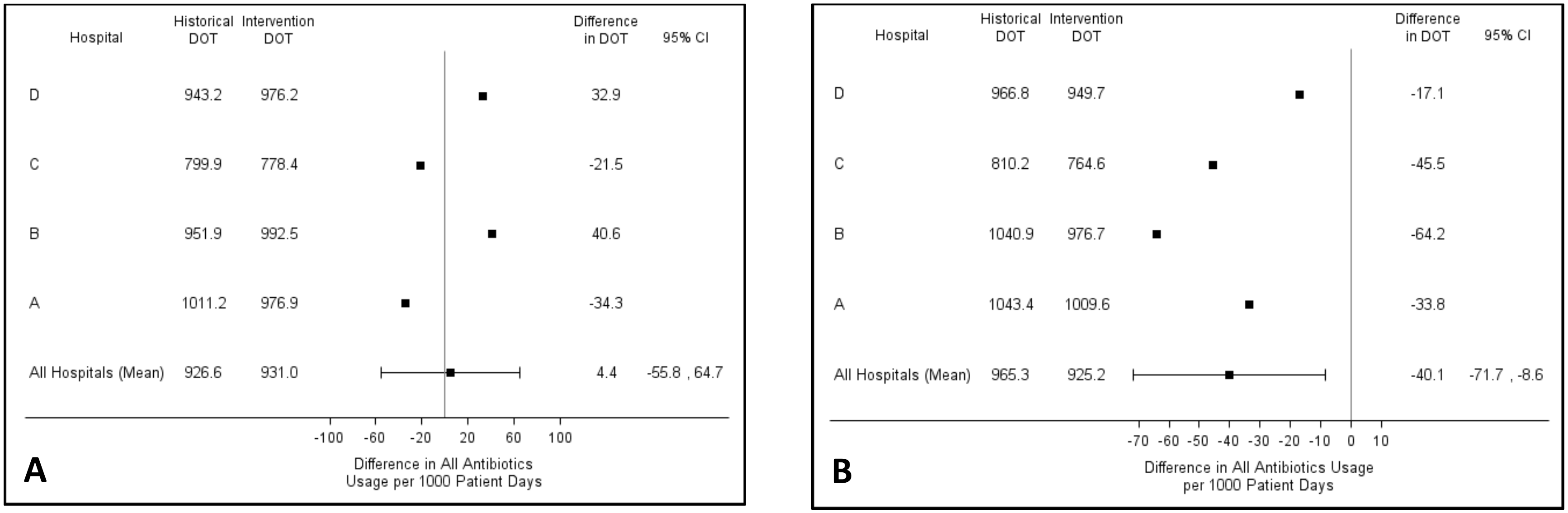

© 2019 Anderson DJ et al. *JAMA Network Open*.

2b. Utilization of all study antimicrobials* among patients who received any antibiotic during the pre-authorization phase (A) and post-prescription audit and feedback (B) interventions

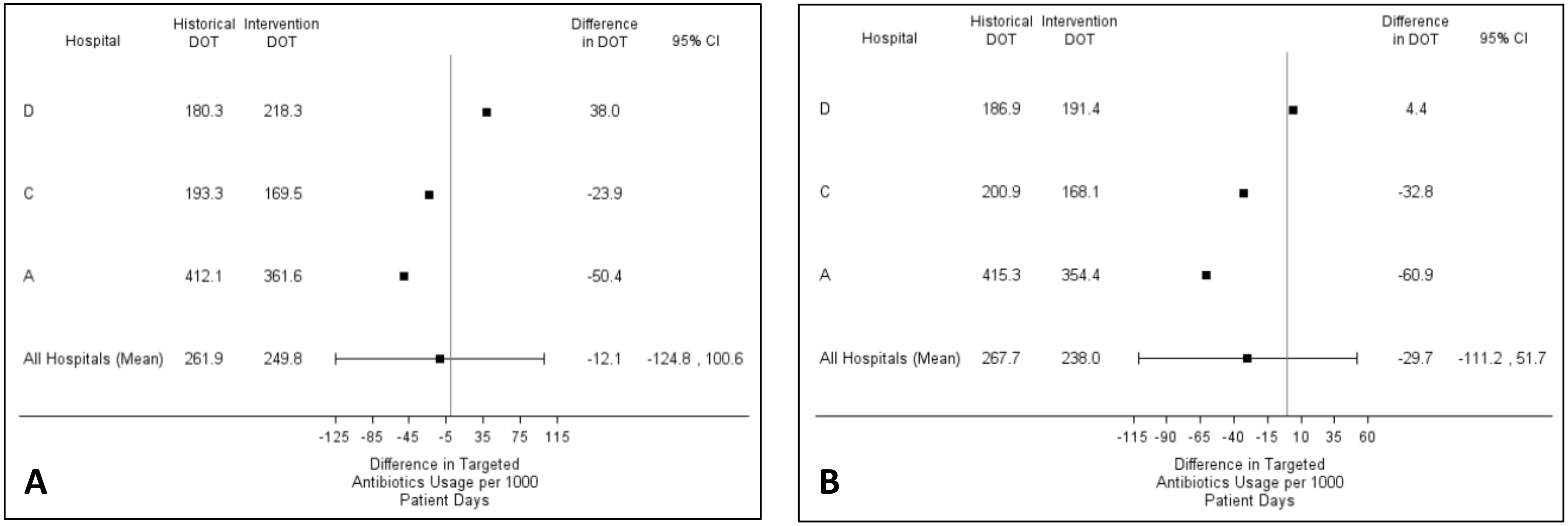

*Hospital B excluded from analysis due to piperacillin-tazobactam shortage during study.

© 2019 Anderson DJ et al. *JAMA Network Open*.

2c. Utilization of vancomycin among patients who received any antibiotic during the pre-authorization phase (A) and post-prescription audit and feedback (B) interventions

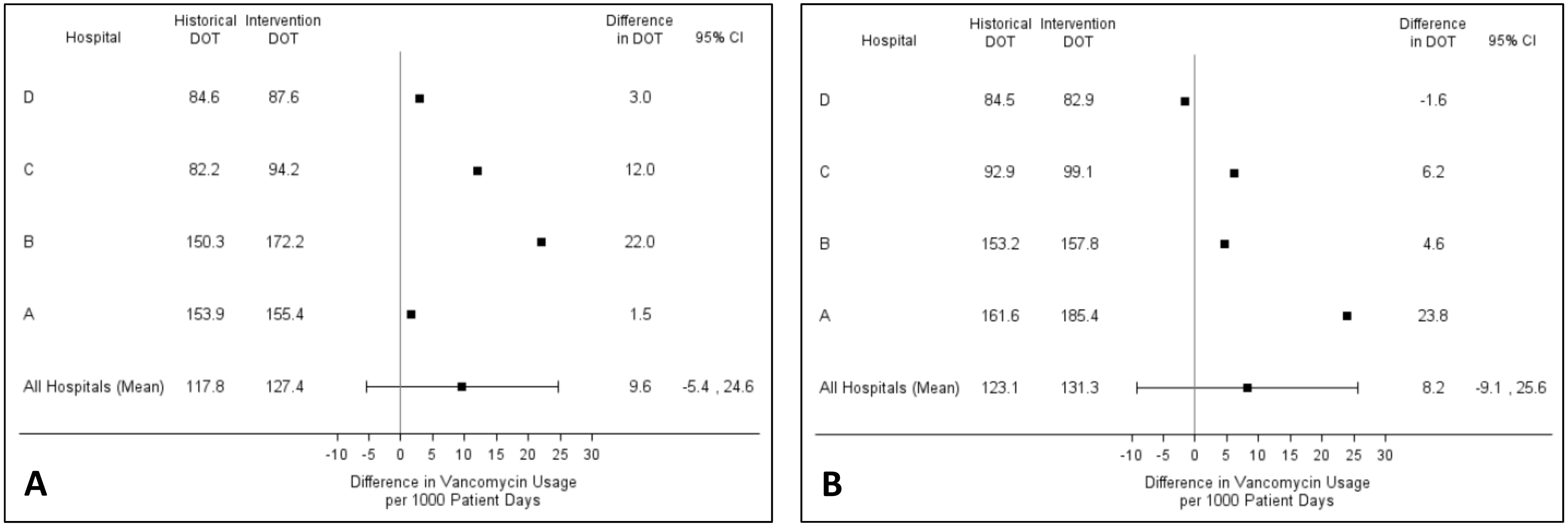

© 2019 Anderson DJ et al. *JAMA Network Open*.

2d. Utilization of piperacillin-tazobactam* among patients who received any antibiotic during the pre-authorization phase (A) and post-prescription audit and feedback (B) interventions

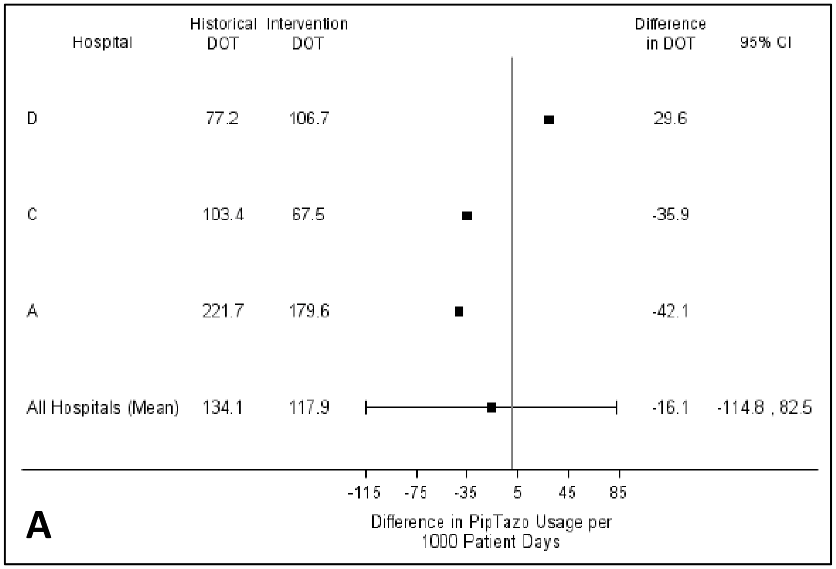

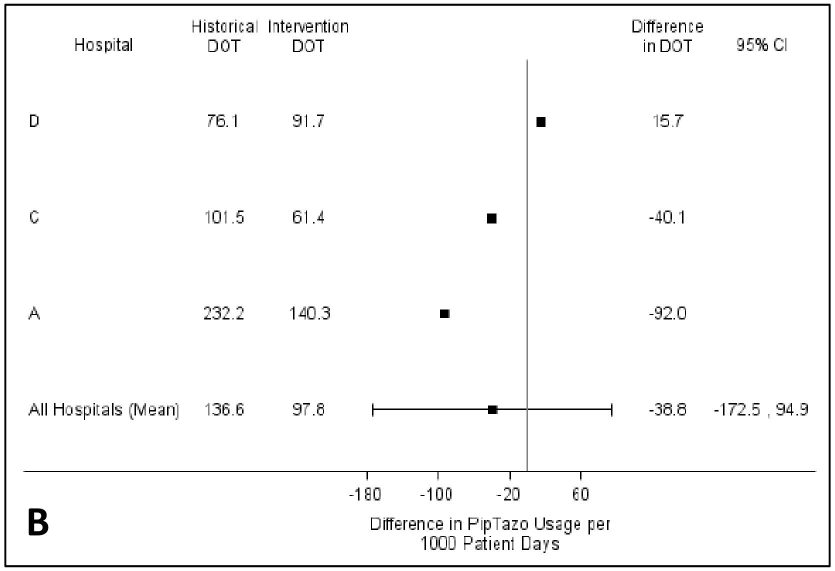

* Hospital B excluded from analysis due to piperacillin-tazobactam shortage during study.

© 2019 Anderson DJ et al. *JAMA Network Open*.

2e. Utilization of anti-pseudomonal carbapenem* among patients who received any antibiotic during the pre-authorization phase (A) and post-prescription audit and feedback (B) interventions

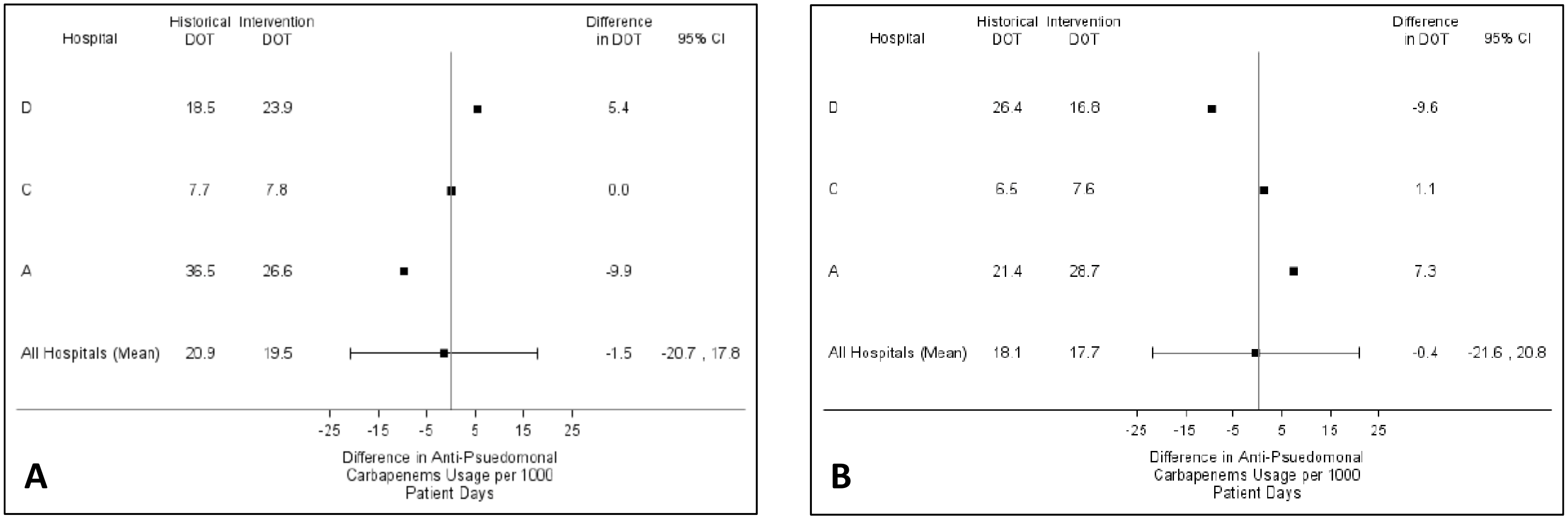

* Hospital B excluded from analysis due to piperacillin-tazobactam shortage during study.

© 2019 Anderson DJ et al. *JAMA Network Open*.

eFigure 3. Changes in Antibiotic Utilization During the 3-Stage Prospective Crossover Study

eFigure 3a. Days of Therapy (DOT)/1,000 patient days for All Study and Non-Study Antibiotics

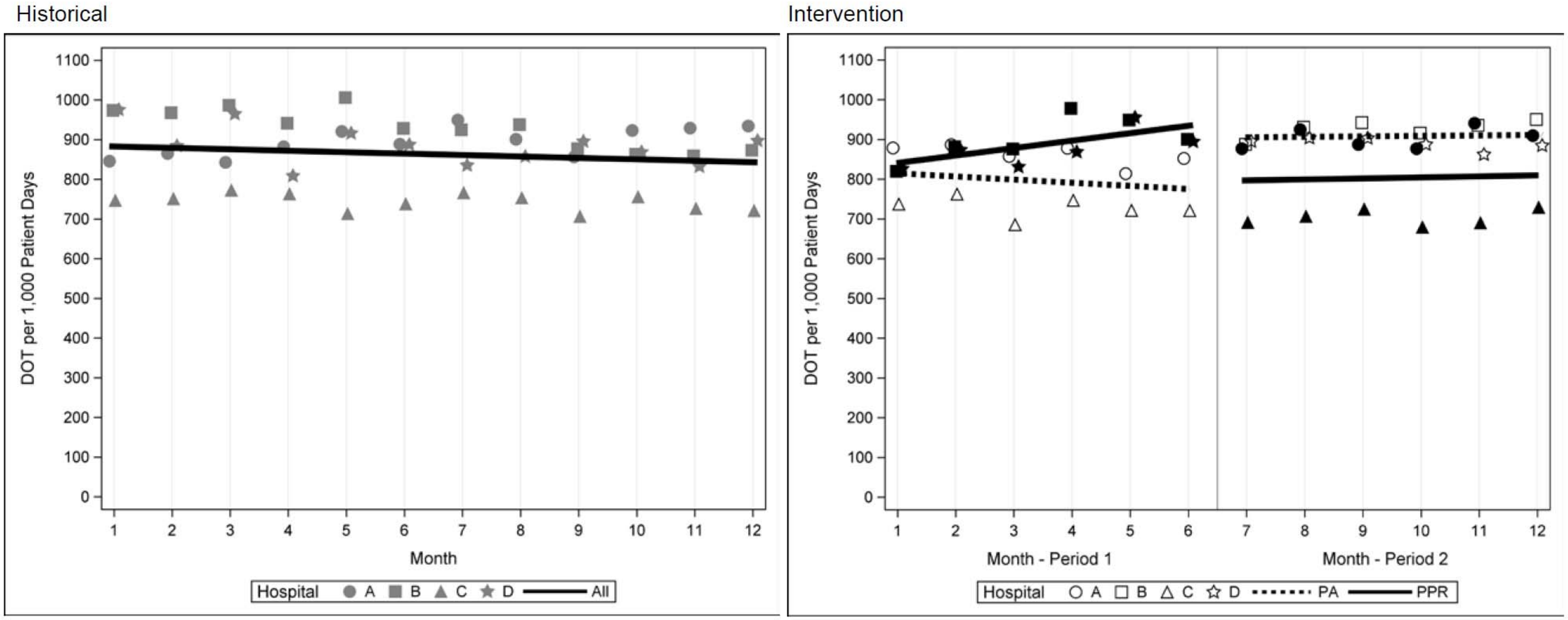

- Grey points indicate Historical
- White points indicate PA
- Black points indicate PPR

PA = modified pre-authorization intervention

PPR = post-prescription audit and review intervention

© 2019 Anderson DJ et al. *JAMA Network Open*.

eFigure 3b. Days of Therapy (DOT)/1,000 patient days for All Study Antibiotics

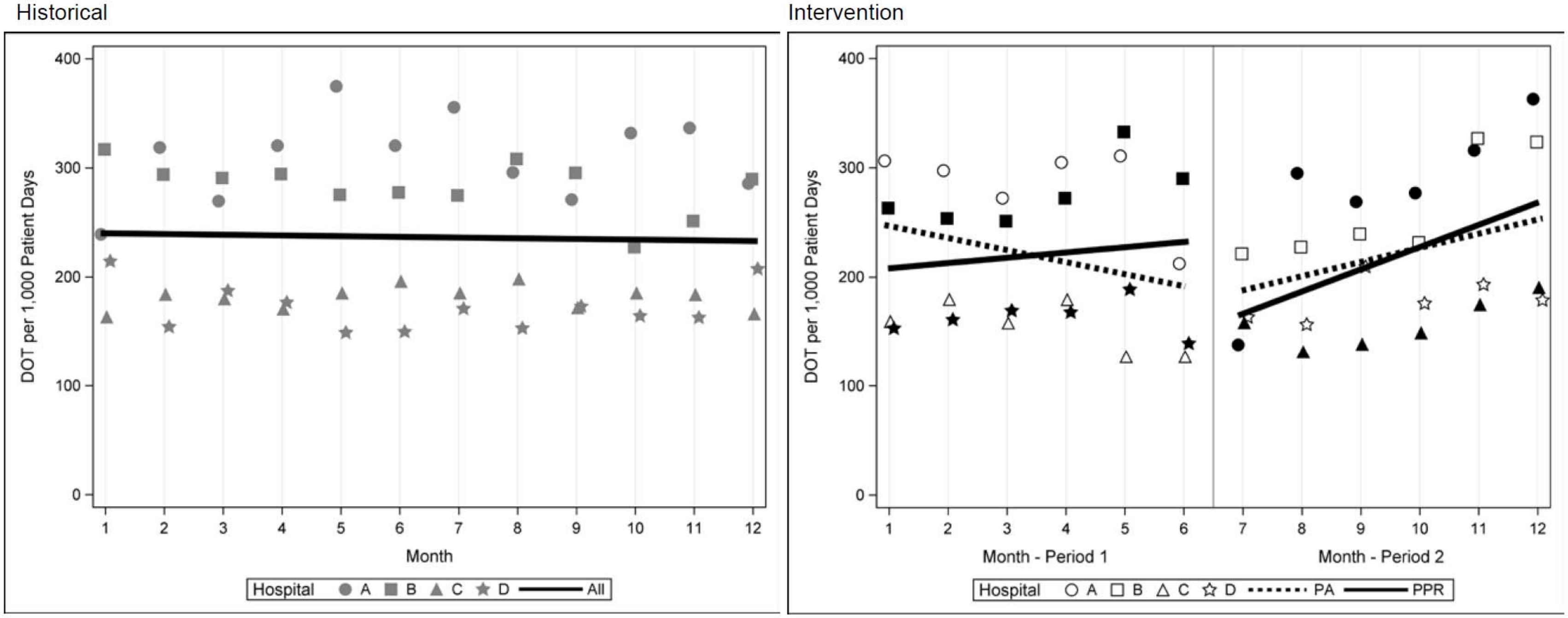

- Grey points indicate Historical
- White points indicate PA
- Black points indicate PPR

PA = modified pre-authorization intervention

PPR = post-prescription audit and review intervention

© 2019 Anderson DJ et al. *JAMA Network Open*.

eTable 1. List of all Study and Non-Study Antimicrobial Agents

**ANTIBIOTIC**
Amikacin
Ampicillin
Aztreonam
Cefazolin
Cefotaxime
Cefotetan
Cefoxitin
Ceftazidime
Ceftriaxone
Cefuroxime
Ciprofloxacin
Clindamycin
Colistimethate
Dicloxacillin
Doxycycline
Fosfomycin
Minocycline
Nafcillin
Oxacillin
Rifampin
Tetracycline
Tobramycin
Sulfamethoxazole with Trimethoprim
Vancomycin
Amoxicillin with Clavulanate
Cefepime
Daptomycin
Meropenem
Imipenem with Cilastatin
Piperacillin with Tazobactam
Levofloxacin
Ticarcillin with Clavulanate
Doripenem

© 2019 Anderson DJ et al. *JAMA Network Open*.

Quinupristin with Dalfopristin
Moxifloxacin
Gentamicin
Linezolid
Ertapenem
Tigecycline
Ampicillin with Sulbactam
Ceftaroline
Ceftolozane/Tazobactam
Trimethoprim
Gatifloxaxin

© 2019 Anderson DJ et al. *JAMA Network Open*.

eTable 2. Comparison of Historical Baseline vs. Intervention Data—Description of Hospitalizations During Which Patients Received Study or Non-Study Antibiotics*

	**Historical Baseline**<br><br>**N=25,283**<br>**n (%)**	**Pre-Authorization**<br><br>**N=14,195**<br>**n (%)**	**Post-Prescription Audit and Review**<br>**N=13,539**<br>**n (%)**
Age – median (IQR)	60 (39-75)	59 (39-74)	60 (41-74)
Female	10,410 (41)	5,952 (42)	5,556 (41)
Race			
Caucasian	12,542 (50)	6,988 (49)	6,751 (50)
African American	9,266 (37)	5,175 (36)	4,982 (37)
Native American	2,943 (12)	1,720 (12)	1,501 (11)
Non-Hispanic ethnicity	24,457 (97)	13,797 (97)	13,168 (97)

*Study antibiotics included vancomycin, piperacillin-tazobactam, and the formulary anti-pseudomonal carbapenem. See Supplemental Table 1 for full list of non-study antibiotics.

© 2019 Anderson DJ et al. *JAMA Network Open*.

eTable 3. Patient Demographics, Intervention Descriptors and Compliance Data by Study Hospital

	**Hospital A** n (%)	**Hospital B[1]** n (%)	**Hospital C** n (%)	**Hospital D** n (%)
Patient Demographics of Admitted Patients during Study Phases 2 and 3	**N=2,960**	**N=5,086**	**N=9,636**	**N=10,052**
Female	1283 (43)	2083 (41)	3713 (39)	4429 (44)
Race				
Caucasian	1418 (48)	2856 (56)	3523 (37)	5942 (59)
African American	1481 (50)	2134 (42)	2585 (27)	3957 (39)
Native American	19 (1)	2 (<1)	3149 (33)	51 (1)
Non-Hispanic ethnicity	2907 (98)	4853 (95)	9255 (96)	9950 (99)
Intervention Descriptors				
Most common forms of contact with prescriber	**N=402**	**N=627**	**N=947**	**N=716**
Phone call	54 (13)	228 (36)	33 (4)	227 (32)
In person	40 (10)	7 (1)	128 (14)	4 (1)
Chart review only	72 (18)	360 (57)	355 (38)	455 (64)

© 2019 Anderson DJ et al. *JAMA Network Open*.

Intervention on targeted antibiotic during pre-authorization period	**N=129**	**N=349**	**N=628**	**N=350**
Vancomycin	72 (56)	269 (77)	236 (56)	223 (64)
Piperacillin-tazobactam	80 (63)	46 (13)	229 (55)	173 (49)
Carbapenem	9 (7)	134 (38)	22 (5)	46 (13)
Intervention on targeted antibiotic during post-antibiotic prescription review period	**N=273**	**N=278**	**N=319**	**N=366**
Vancomycin	175 (64)	203 (73)	103 (66)	219 (62)
Piperacillin-tazobactam	138 (51)	168 (60)	82 (53)	183 (52)
Carbapenem	28 (10)	21 (8)	3 (2)	34 (10)
Recommendation followed (if given)	**N=93**	**N=327**	**N=228**	**N=262**
Yes, all	73 (78)	140 (43)	155 (68)	167 (64)
Yes, some	6 (6)	65 (20)	27 (12)	41 (16)
No	14 (15)	122 (37)	46 (20)	54 (21)

1 – Hospital B had a shortage of piperacillin-tazobactam during the study

© 2019 Anderson DJ et al. *JAMA Network Open*.

eTable 4. Impact of Core Stewardship Interventions on Days of Therapy (DOT) of Antimicrobials per 1,000 Patient Days Among All Patients Who Received Study or Non-Study Antimicrobials Compared to Matched Historical Baseline

	**Pre-Authorization**<br>**Difference in DOT/1,000 patient-days compared to historical baseline (95% CI)**	**Post-Prescription Audit and Review**<br>**Difference in DOT/1,000 patient-days compared to historical baseline (95% CI)**
All study and non-study antimicrobials	4.4 [-55.8 to 64.7]	-40.1 [-71.7 to -8.6]
All study antimicrobials[a]	-12.1 [-124.8 to 100.6]	-29.7 [-111.2 to 51.7]
Vancomycin	9.6 [-5.4 to 24.6]	8.2 [-9.1 to 25.6]
Piperacillin-tazobactam[a]	-16.1 [-114.8 to 82.5]	-38.8 [-172.5 to 94.9]
Anti-pseudomonal carbapenem[a]	-1.5 [-20.7 to 17.8]	-0.4 [-21.6 to 20.8]

A - Hospital B excluded from analysis due to piperacillin-tazobactam shortage during the study

© 2019 Anderson DJ et al. *JAMA Network Open*.

eAppendix 2. Guidance for Use of Targeted Antibiotics

The study team provided documents to pharmacists at each study hospital that provided general guidance about the use of targeted antibiotics during the Pre-Authorization and Post-Antimicrobial Prescription Review study phases. These guidance documents, which represented our best guidance at the beginning of the study in 2014, are included here for reference:

General Guidance for Use of Targeted Antimicrobials – PRE-AUTHORIZATION

GENERAL ADVICE

Risk factors for MRSA/MDRO pathogens include:

1. Hospitalization in the past 90 days
2. Reside in a long term care facility (LTCF) in the past year
3. Received antibiotics or chemotherapy in the past 30 days
4. Currently requiring hemodialysis for end stage renal disease
5. In the hospital more than 2 days at the time of infection

Avoid giving advice on duration of therapy.

Focus on appropriateness of drug as empiric treatment.

VANCOMYCIN

Question(s) to ask the provider: Do you really need MRSA coverage?

Be prepared to give Vancomycin alternatives if you deem therapy to be inappropriate.

**Indication**	**Explanation**
**PNEUMONIA[1,2]**	
Community Acquired Pneumonia (CAP)	**Avoid:** Unlikely to be appropriate, even if headed to ICU.<br>**Use:** Appropriate if high suspicion for CA-MRSA pneumonia.[2] Should have radiographic evidence of severe/cavitary (abscess) pneumonia and history of skin abscesses to have suspicion of CA-MRSA.
HCAP/HAP	**Avoid:** Inappropriate if no risk factors for HCAP/HAP.<br>**Use:** Likely to be appropriate in almost all scenarios since these patients are at risk for MDR. Key is confirming that patient meets the criteria for "healthcare-associated" infection or HAP (**see MRSA/MDRO risk factors**)
Aspiration	**Avoid:** Inappropriate unless risk factors/onset is consistent with HCAP/HAP. Main pathogens are gram-negatives and anaerobes.<br>**Use:** Appropriate if patient has risk factors/onset is consistent with HCAP/HAP.

© 2019 Anderson DJ et al. *JAMA Network Open*.

	Try to remind prescriber that may be pneumonitis instead of pneumonia and if resolves quickly only short (3d) course needed.
**UTI/ PYELONEPHRITIS /UROSEPSIS[3,4]**	Avoid: Unlikely to be appropriate for "uncomplicated UTI" or pyelonephritis, particularly in absence of a catheter.<br><br>Use: Appropriate if uncomplicated UTI or pyelonephritis with h/o urologic procedure or instrumentation, ampicillin-resistant Enterococcus UTI, or MRSA UTI. If catheter present, empiric vancomycin may be appropriate in short term (but controversial).
**MENINGITIS**	Avoid: Inappropriate if aseptic meningitis on CSF (i.e. low WBC count, nl glucose/protein, lymphocytic predominance).<br><br>Use: Appropriate for all empiric treatment of all cases of suspected bacterial meningitis due to the possibility of infection due to beta-lactam resistant *S. pneumonia.* This organism is not very common.<br><br>***Ask if have obtained LP/CSF cell counts. If this diagnosis is being considered strongly enough to initiate antibiotics, then MUST have diagnostic testing.***
**SURGICAL SITE INFECTION (SSI)[5]**	Use: Appropriate in almost all instances.<br><br>Avoid: Exceptions may be SSIs following GU or GI surgeries.
**SKIN AND SOFT TISSUE INFECTION (SSTI)[5]**	Avoid: Inappropriate if non-purulent cellulitis (no abscess), unless has one of the following: severe infection (in ICU or hypotension) or immunocompromised.<br><br>Use: Appropriate if pus/purulent infection, signs of deeper infection (bullae, skin sloughing), severe sepsis, or concern for necrotizing fasciitis.
**DIABETIC FOOT INFECTIONS (DFI)[6]**	***Note: Many diabetic foot ulcers are NOT infected. Ask prescriber if there is purulent drainage OR evidence of at least 2 signs of infection (erythema, warmth, tenderness, pain, or induration).***<br><br>Avoid: If none of above, not appropriate; only local wound care and off-loading is needed.<br><br>Use: Appropriate if h/o MRSA or if severe/systemic symptoms. MDs may argue for it and probably ok in most instances of empiric treatment of DFI.
**BACTEREMIA *(implies blood culture already positive; if not, see "empiric sepsis)***	Avoid: If GNR in blood – most likely not needed, though MD may argue that need broad coverage until cultures finalized if severe sepsis.<br><br>Use: Appropriate if GPC in blood culture. If species available, then need to know susceptibilities. MRSA and coagulase-negative Staph will be the most common organisms from blood that require vancomycin.
**EMPIRIC SEPSIS**	Avoid: May not be appropriate for patients without MRSA risk factors, but prescriber may want medication depending on how "sick" the patient is. Probably reasonable to give for patients headed to ICU.

© 2019 Anderson DJ et al. *JAMA Network Open*.

	**Use:** Appropriate for patients with MRSA risk factors.
**PENICILLIN ALLERGY**	**Avoid:** Not appropriate if patient does not meet criteria to receive vancomycin AND does not have type I hypersensitivity/anaphylaxis to penicillins. If cephalosporins may be given to these patients safely, then vancomycin is not appropriate.<br><br>**Use:** Appropriate if patient meets criteria to receive vancomycin OR they have a type I hypersensitivity reaction to penicillin.

PIPERACILLIN-TAZOBACTAM

Question(s) to ask the provider: **(1) Do you really need Pseudomonas coverage?**

(2) Do you really need anaerobic coverage?

P/T is a reasonable choice for patients with severe sepsis/septic shock and healthcare exposure.

For similar coverage, probably need 2 drugs:

1. Cipro + Metro
2. Ceftaz or CFP + Clinda
3. Ceftaz or CFP + Metro
4. Cipro + Clinda
5. Moxi (some anaerobic coverage)

Amp-sulbactam is not a good alternative for abdominal or HCAP/HAP coverage as not very effective against E. coli and other common GNR (and doesn't cover PA); however it is ok for empiric coverage of ENT infections and in some cases of CAP.

**Indication**	**Explanation**
**PNEUMONIA**[1,2]	
CAP	**Avoid:** Not appropriate
HCAP/HAP	**Avoid:** Inappropriate if patient does not meet criteria for HCAP/HAP.<br><br>**Use:** Very likely to be appropriate in almost all scenarios. Key is confirming that patient meets the criteria for "healthcare-associated" infection or HAP (***See General comments above***)
Aspiration	**Avoid:** Inappropriate if alternative agents can be given (see below).<br><br>**Use:** Probably appropriate, but consider alternative agents mentioned above (e.g., Cipro + Clinda or if community-acquired and little PsA risk: moxi or amp/sulbactam).<br><br>***Try to remind prescriber that may be pneumonitis instead of pneumonia and if resolves quickly only short (3d) course needed.***

© 2019 Anderson DJ et al. *JAMA Network Open*.

**UTI/ PYELONEPHRITIS /UROSEPSIS**[3,4]	Avoid: Not appropriate for "uncomplicated UTI" or pyelonephritis, particularly in absence of a catheter<br><br>Use: May be appropriate if catheter present, but coverage is more than needed. Consider alternatives such as ceftriaxone or ciprofloxacin, as anaerobic coverage not necessary.
**MENINGITIS**	Avoid: In general, not appropriate for empiric treatment of suspected community-onset bacterial meningitis. (For meningitis following a neurosurgical procedure, anti-pseudomonal drugs of choice would be cefepime, ceftazidime, or meropenem.)<br><br>***If this diagnosis is being considered strongly enough to initiate antibiotics, then MUST have diagnostic testing.***
**SURGICAL SITE INFECTION (SSI)**[5]	Use: SSI implies healthcare exposure, so may be appropriate. While some resulting risk of *P. aeruginosa* infection, PA is a rare SSI pathogen. In particular, may be reasonable for empiric choice following GI, GU, or GYN but guidelines suggest cephalosporin + metronidazole.<br><br>***Push for microbiologic diagnosis whenever possible (e.g. drain abscesses to get micro data).***
**SKIN AND SOFT TISSUE INFECTION (SSTI)**[5]	Avoid: Inappropriate in patients who do not have "severe" infection.<br><br>Use: Appropriate in patients with "severe" skin and soft tissue infection, including in ICU, hypotension (signs of severe sepsis/septic shock), or immunocompromised.
**DIABETIC FOOT INFECTIONS (DFI)**[6]	***Note: Many diabetic foot ulcers are NOT infected. Ask prescriber if there is purulent drainage OR evidence of at least 2 signs of infection (erythema, warmth, tenderness, pain, or induration).***<br><br>Avoid: If none of the above, not appropriate. Not appropriate if mild to moderate infection and no recent antibiotics. Guidelines suggest only coverage of GPC.[6]<br><br>Use: If severe infection (see above), then appropriate to provide "broad spectrum coverage". As before, alternative regimens will be just as effective. Coverage for Pseudomonas only really necessary if appropriate risk factors or history of Pseudomonas infection.
**BACTEREMIA *(implies blood culture already positive; if not, see "empiric sepsis)***	Avoid: If GPC in blood – most likely not appropriate, though MD may argue that need broad coverage until cultures finalized if severe sepsis.<br><br>Use: If GNR in blood culture, appropriate empiric choice until susceptibilities available.
**EMPIRIC SEPSIS**	Avoid: May not be appropriate for patients without Pseudomonas risk factors, but probably reasonable for patients with severe sepsis/ septic shock (i.e., prescriber may want medication depending on how "sick" the patient is).<br><br>Use: Appropriate for patients with Pseudomonas risk factors. Probably reasonable to give for patients headed to ICU.

© 2019 Anderson DJ et al. *JAMA Network Open*.

**INTRA-ABDOMINAL INFECTION[7]**	***Guidelines separate recommendations into several different types of intra-abdominal infections (e.g., biliary, diverticulitis, peritonitis, etc).***<br><br>**Avoid:** Inappropriate if not severe infection (i.e., hypotension or ICU). Alternatives such as cefazolin + metronidazole are likely as effective.<br><br>**Use:** Appropriate if infection is severe or followed a procedure (i.e, healthcare-associated). Though alternatives are likely as effective (e.g., ceftazidime and metronidazole).

ANTI-PSEUDOMONAL CARBAPENEM (Imipenem, Meropenem, Doripenem)

Question(s) to ask the provider: **(1) Do you really need Pseudomonas coverage?**

(2) Do you really need anaerobic coverage?

(3) Why not use P/T instead of a carbapenem?

There are few scenarios in which empiric use of a carbapenem is appropriate. If an antibiotic with as much broad coverage as a carbapenem is required, then P/T would be a reasonable substitute in the vast majority of cases.

Therefore, the same guidance provided above for use of P/T can be used when adjudicating the use of the carbapenem. BUT, when adjudicating, try to steer therapy to P/T (instead of the carbapenem) whenever possible.

If the prescriber continues to want the carbapenem, ask "why do you want the carbapenem instead of P/T?". If the answer is "broader coverage," you can point out that P/T has better coverage for PA (per antibiogram) than carbapenem in ALL FOUR STUDY HOSPITALS.

Avoid: Use of the carbapenem is not appropriate when P/T is a reasonable alternative.

Use: Carbapenems are appropriate in the following scenarios

1. History of ESBL infection
2. History of infection with a highly drug-resistant organism with proven resistance to P/T and susceptibility to the carbapenem
3. Septic shock in highly immunocompromised patients (e.g., febrile neutropenia or transplant patients)
4. Post-operative infection following neurosurgical procedure

Sometimes Use:

1. Pancreatitis
    a. Carbapenems are frequently requested for empiric therapy for pancreatitis.
    b. Technically, empiric antibiotics are not recommended in patients with acute pancreatitis, regardless of the type (interstitial or necrotizing) or disease severity (mild, moderately severe, or severe)[8]
    c. While CT scans aren't required for diagnosis of pancreatitis, they are useful for diagnosing "necrotizing" pancreatitis.

© 2019 Anderson DJ et al. *JAMA Network Open*.

    1. There is no correlation, however, between the extent of necrosis and the risk of infection.
  d. If infected necrosis is suspected due to fever, increased WBC, and CT findings, carbapenems are appropriate antibiotics.
  e. BUT, you can also suggest that they pursue FNA of necrotic areas to confirm infection.

eReferences 1:

1. Guidelines for the management of adults with hospital-acquired, ventilator-associated, and healthcare-associated pneumonia. American journal of respiratory and critical care medicine 2005;171:388-416.

2. Mandell LA, Wunderink RG, Anzueto A, et al. Infectious Diseases Society of America/American Thoracic Society consensus guidelines on the management of community-acquired pneumonia in adults. Clinical infectious diseases : an official publication of the Infectious Diseases Society of America 2007;44 Suppl 2:S27-72.

3. Gupta K. et al. International Clinical Practice Guidelines for the Treatment of Acute Uncomplicated Cystitis and Pyelonephritis in Women: A 2010 Update by the Infectious Diseases Society of America and the European Society for Microbiology and Infectious Diseases. Clin Infect Dis 2011;52(5):e103–e12.

4. Nicolle LE et al. Infectious Diseases Society of America Guidelines for the Diagnosis and Treatment of Asymptomatic Bacteriuria in Adults. Clin Infect Dis 2005; 40: 643-54.

5. Stevens DL et al. Practice Guidelines for the Diagnosis and Management of Skin and Soft Tissue Infections: 2014 Update by the Infectious Diseases Society of America. Clin Infect Dis 2014; doi: 10.1093/cid/ciu296.

6. Lipsky BA et al. 2012 Infectious Diseases Society of America Clinical Practice Guideline for the Diagnosis and Treatment of Diabetic Foot Infections. Clin Infect Dis 2012;54(12):132-173.

7. Solomkin JS et al. Diagnosis and management of complicated intra-abdominal infection in adults and children: guidelines by the Surgical Infection Society and the Infectious Diseases Society of America. *Clin Infect Dis.* 2010;50(2):133-64.

8. Tenner S et al. *American College of Gastroenterology guideline: management of acute pancreatitis. Am J Gastroenterol 2013.*

© 2019 Anderson DJ et al. *JAMA Network Open*.

eAppendix 3. General Guidance for Use of Targeted Antimicrobials – POST-PRESCRIPTION REVIEW

<u>GENERAL ADVICE</u>

Key component of review at this point is data that have accumulated for the past ~72 hours on therapy

1) Clinical picture improved? *This scenario cuts both ways*. Improvement may provide the opportunity to de-escalate and/or convert to PO therapy. BUT, clinicians may also argue along the lines of "why change when patient improved on this therapy."
2) Microbiology data are KEY. Act on both positive AND negative cultures.

Can give advice on duration in this setting, but need to temper with statements like "if things continue to progress well," and/or "according to X guidelines, typical duration of therapy is X days."

1) We want to be careful not to provide clinical recommendations for very complicated patients.
2) Duration recommendations provided below are intended to provide general guidance.

Risk factors for MRSA/MDRO pathogens include:

1. Hospitalization in the past 90 days
2. Reside in a long term care facility (LTCF) in the past year
3. Received antibiotics or chemotherapy in the past 30 days
4. Currently requiring hemodialysis for end stage renal disease
5. In the hospital more than 2 days at the time of infection

Focus on appropriateness of drug as <u>directed</u> treatment. Seek alternative, narrower agents to complete therapy when antibiotics are indicated (but not the drug under review).

Question(s) to ask the provider:

1) Do we need to continue X to complete therapy?
2) Can we de-escalate therapy? [Is there a more narrow and targeted drug that we can use?]
3) Can patient tolerate an oral regimen? (*if appropriate for the infection being treated*)

If complicated patient and ID consult service available at your hospital, it is reasonable to recommend ID consultation.

<u>VANCOMYCIN</u>

Question(s) to ask the provider: Do you really need MRSA coverage?

Be prepared to give vancomycin alternatives if you deem therapy to be inappropriate.

Indication	Explanation
**PNEUMONIA[1,2]**	

© 2019 Anderson DJ et al. *JAMA Network Open*.

Community Acquired Pneumonia (CAP)/HCAP/HAP	**Avoid:** Not appropriate if respiratory culture was good quality and results DON'T show MRSA, even if in ICU.<br><br>**Use:** Appropriate for microbiologically-diagnosed MRSA pneumonia. Per 2007 guidelines, vanco only appropriate if MRSA has been isolated in culture specimens.[2]<br><br>**Duration**: If treating MRSA pneumonia, 7 days if no cavity or bacteremia.
Aspiration	**Avoid:** Not appropriate unless there is microbiologic evidence of MRSA. Main pathogens are gram-negatives and anaerobes.<br><br>**Use:** Appropriate for microbiologically-diagnosed MRSA pneumonia.<br><br>**Duration**: If treating MRSA pneumonia, 7 days if no cavity nor bacteremia<br><br>***If CXR findings quickly resolve, most likely pneumonitis and antibiotics not necessary > 3 days.***
**UTI/ PYELONEPHRITIS /UROSEPSIS[3,4]**	**Avoid:** Not appropriate for asymptomatic bacteriuria, contaminated specimen, or culture with growth other than MRSA.<br><br>***Should have urinalysis and urine cx to review. While urinalysis may grow bacteria (even MRSA) and have WBC, keys to appropriateness are 1) was patient symptomatic when therapy was started? and 2) is the specimen contaminated?***<br><br>***If patient was asymptomatic at the time of diagnosis, antibiotics are NOT appropriate. Pyuria frequently occurs with asymptomatic bacteriuria and does not, by itself, indicate the presence of an infection or need for antibiotics.***<br><br>***If specimen is contaminated (look for squams on U/a), antibiotics may not be appropriate. Treatment decisions CANNOT be made based on contaminated urine specimens. Another specimen needs to be sent if clinical suspicion of UTI remains.***<br><br>**Use:** Appropriate for treatment of ampicillin-resistant Enterococcus UTI, MRSA UTI, MRSA asymptomatic bacteriuria in a pregnant patient.<br><br>***If Vanco is appropriate for UTI pathogen but infection is otherwise uncomplicated, work with clinician to make a rec for completion of therapy that does not include IV vanco (e.g. switch to orals for completion of therapy?)***<br><br>**Duration**:<br>• UTI 3-7 days, depending on if uncomplicated vs. complicated or CAUTI<br>• Pyelo 7-14 days
**MENINGITIS**	**Avoid:** Not appropriate if aseptic meningitis on CSF or no evidence of CTX-R *S. pneumoniae* (but check allergies)<br><br>**Use:** Appropriate for MRSA-confirmed meningitis (very rare).<br><br>***Ask if have obtained LP/CSF cell counts. If this diagnosis is strongly suspected (and receiving antibiotics as a result), then MUST have diagnostic testing. Advocate for an LP even if patient has already received antimicrobial therapy***

© 2019 Anderson DJ et al. *JAMA Network Open*.

	**Duration**: 14 days for CTX-R *S. pneumoniae*
**SURGICAL SITE INFECTION (SSI)**[5]	**Avoid**: Not appropriate if culture growing GNR or GPC with susceptibility to β-lactam (e.g., MSSA or Strep). Not appropriate if cultures negative and no hardware (e.g., prosthetic joint) in place.<br><br>**Use:** Appropriate if culture is growing GPC (but susceptibilities pending) or MRSA. If no cultures, but suspicion for true infection high, probably appropriate to continue.<br><br>***If no cultures were obtained, provide education about importance of culture data to guide antimicrobial therapy.***<br><br>**Duration**: Depends on extent of SSI<br>• Superficial SSI - 5-10 days<br>• More invasive SSI - **Do not provide duration recommendation** (duration depends on need for I&D, type of procedure, implant)
**SKIN AND SOFT TISSUE INFECTION (SSTI)**[5]	**Avoid**: Not appropriate if cultures negative for MRSA.<br><br>**Use:** Acceptable to continue if no cultures but skin infection had signs of deeper infection (bullae, skin sloughing), severe sepsis, or concern for necrotizing fasciitis. Appropriate if non-purulent cellulitis coupled with a) severe infection (ICU or hypotensive), b) immunocompromised, or c) worsening or no improvement in clinical syndromes.<br><br>***If "deep" infection but no culture data, push for I&D to obtain culture data, even if already on antibiotics***<br><br>***Note that erythema of skin does not regress quickly, even with appropriate therapy***<br><br>**Duration:** 5 days for uncomplicated SSTI. May require 2 or more weeks if multiple I&Ds required (signs of severe sepsis/septic shock) OR immunocompromised.
**DIABETIC FOOT INFECTIONS (DFI)**[6]	***Note: Many diabetic foot ulcers are NOT infected. Ask prescriber if there was purulent drainage OR evidence of at least 2 signs of infection (erythema, warmth, tenderness, pain, or induration) at the time of diagnosis.***<br><br>**Avoid:** If none of above, not appropriate; only local wound care and off-loading is needed. Not appropriate if oral option is reasonable, systemic symptoms resolved, and no deep infection (i.e, osteo).<br><br>**Use:** Appropriate if culture positive for MRSA and signs of infection. Appropriate if h/o MRSA and signs of infection. Appropriate if severe/systemic symptoms are unresolved.<br><br>***Push for culture data, preferably from "good" specimens (ie, OR specimen, deep culture of bone if suspected osteo, or collection of pus; not a simple wound swab). On the other hand, wound swabs that DO NOT grow MRSA can be used to recommend stopping Vancomycin.***<br><br>**Duration:** Depends on type of infection<br>• Mild DFI – switch to orals (e.g. tmp/smx or doxycycline) to complete 1-2 weeks

© 2019 Anderson DJ et al. *JAMA Network Open*.

	• Moderate to severe DFI – 2-3 weeks, though duration depends on need for I&D, bone involvement (confirmed osteomyelitis requires debridement followed by 6 weeks of IV antibiotics), clinical improvement
**BACTEREMIA**	<u>**General**</u>:<br>• Determine the primary source of infection to determine best antibiotic and clinical management. ID consultation may be required.<br>• Duration recommendations (in general) will need to be made after the full diagnostic workup is complete.<br>o Consider if the primary source is potentially polymicrobial (e.g. abdominal or DFI), even if only a single pathogen is identified in the blood.<br>▪ If so, then broader antibiotic coverage for the polymicrobial infection may be clinically required.<br>• The guidelines below are based on the assumption that the primary infectious source includes *only* the identified pathogen in the blood.<br>**<u>Avoid</u>**: <u>Not appropriate</u> if MSSA, nafcillin-S CoNS, Streptococci, Amp-S Enterococci, Vanc-I or Vanc-R Enterococci, any GNR, or fungal pathogen in blood.<br><br>**<u>Use:</u>** <u>Appropriate</u> if blood culture grows MRSA, nafcillin-R CoNS, CoNS with no susceptibilities provided (assume nafcillin-R), or Amp-R Enterococci.<br><br>***A single blood culture with CoNS, Viridans Group Strep, or Enterococcus most likely represents a contaminated blood culture. In this setting, recommend that MD order repeat blood cultures. If follow-up cultures are negative, stop vancomycin in most cases.***<br><br>**<u>Duration:</u>** Treatment duration of MRSA BSI is too complicated to provide duration.
**EMPIRIC SEPSIS**	<u>**General:**</u><br>• Determine the primary source of infection to determine best antibiotic and clinical management.<br>o If infection source has been determined, refer to sections for that specific type of infection.<br>• If infection source remains unclear, then determine if the patient has clinically improved on empiric therapy.<br>o Determine if patient has risk factors for MRSA infection and the working clinical diagnosis or most likely source.<br>***If source not determined,***<br><br>**<u>Avoid:</u>** <u>Not appropriate</u> if no MRSA RF and clinically improving. <u>May not be appropriate</u> if no MRSA RF but not clinically improving. If unwilling to stop, suggest plan to slowly remove antibiotics. Negative cultures can be used to suggest stopping vanco. Alternatively, may suggest they obtain an ID consult if available.<br><br>**<u>Use:</u>** <u>Appropriate</u> if microbiological specimen suggests MRSA infection.<br><br>**<u>Duration:</u>** Do not provide treatment duration recommendations for sepsis of unclear etiology.

© 2019 Anderson DJ et al. *JAMA Network Open*.

**PENICILLIN ALLERGY**	**Avoid:** Not appropriate if patient does not meet criteria to receive vancomycin AND does not have type I hypersensitivity/anaphylaxis to penicillins. If cephalosporins may be given to these patients safely, then vancomycin is not appropriate.<br><br>**Use:** Appropriate if patient meets criteria to receive vancomycin OR they have a type I hypersensitivity reaction to penicillin.

© 2019 Anderson DJ et al. *JAMA Network Open*.

PIPERACILLIN-TAZOBACTAM or ANTI-PSEUDOMONAL CARBAPENEM

Approach to determine appropriateness of P/T and CP is generally the same.

Question(s) to ask the provider: **(1) Do you really need Pseudomonas coverage?**

(2) Do you really need anaerobic coverage?

(3) If meets criteria for CP, is P/T an option?

For similar coverage, probably need 2 drugs:

1. Cipro + Metro
2. Ceftaz or CFP + Clinda
3. Ceftaz or CFP + Metro
4. Cipro + Clinda
5. Moxi (some anaerobic coverage)

Carbapenems may be preferable to P/T in the following scenarios:

1. History of ESBL infection
2. History of infection with a highly drug-resistant organism with proven resistance to P/T and susceptibility to the carbapenem
3. Septic shock in highly immunocompromised patients (e.g., febrile neutropenia or transplant patients)
4. Post-operative infection following neurosurgical procedure

**Indication**	**Explanation**
**PNEUMONIA[1,2]**	
CAP	**Avoid:** Not appropriate unless microbiology data surprisingly demonstrates resistant organism that requires P/T or CP.
HCAP/HAP	**Avoid:** Not appropriate if respiratory culture obtained but results DON'T show Pseudomonas or other resistant GNR.<br>***If culture negative or grows OPF, switch to alternative, narrower agent, if possible (e.g, FQ or cephalosporin).***<br>**Use:** Appropriate if no culture data but has HAP/HCAP (i.e., ongoing empiric treatment). Appropriate if culture with MDR that requires P/T or CP (i.e., no alternative agents like ciprofloxacin).<br>***Provide education about importance of microbiological data to guide antimicrobial therapy.***<br>**Duration:** Treat for 7 days unless treating non-fermenting GNR (e.g. Pseudomonas, AB, steno). If non-fermenting GNR, then treat for 14 days.
Aspiration	**Avoid:** Not appropriate if alternative agents are more appropriate.

© 2019 Anderson DJ et al. *JAMA Network Open*.

	Use: Probably appropriate, but consider alternative agents mentioned above (e.g., Cipro + Clinda or if community-acquired and little PsA risk: moxi or amp/sulbactam).<br><br>***Try to remind prescriber that may be pneumonitis instead of pneumonia and if resolves quickly only short (3d) course needed.***
**UTI/ PYELONEPHRITIS /UROSEPSIS[3,4]**	Avoid: Not appropriate for asymptomatic bacteriuria, contaminated specimen, or culture with growth other than highly-resistant GNR.<br><br>***Should have urinalysis and urine cx to review. While urinalysis may grow bacteria have WBC, keys to appropriateness are 1) was patient symptomatic when therapy was started? and 2) is the specimen contaminated?***<br><br>***If patient is asymptomatic, antibiotics are NOT appropriate. Pyuria frequently occurs with asymptomatic bacteriuria and does not, by itself, indicate the presence of an infection or need for antibiotics.***<br><br>***If specimen is contaminated (look for squams on U/a), antibiotics may not be appropriate. Treatment decisions CANNOT be made based on contaminated urine specimens. So, another specimen needs to be sent if clinical suspicion of UTI remains.***<br><br>Use: Appropriate for UTI/pyelo/urosepsis with highly-resistant GNR.<br><br>***If P/T or CP appropriate for UTI pathogen but otherwise uncomplicated infection, work with clinician to make a rec for completion of therapy that does not include IV abx (e.g. switch to orals for completion of therapy?)***<br><br>Duration:<br>• UTI 3-7 days, depending on if uncomplicated vs. complicated or CAUTI<br>• Pyelo 7-14 days
**MENINGITIS**	Avoid: Not appropriate if aseptic meningitis on CSF or no evidence of meningitis due to a highly-resistant GNR<br><br>Use: Appropriate for meningitis due to highly-resistant GNR (very rare). Carbapenem is probably preferable to P/T in this scenario.<br><br>***If this diagnosis is strongly suspected (and receiving antibiotics as a result), then MUST have diagnostic testing. Advocate for an LP even if patient has already received antimicrobial therapy***<br><br>Duration: 3 weeks for GNR meningitis
**SURGICAL SITE INFECTION (SSI)[5]**	Avoid: Not appropriate if cultures do not grow highly resistant GNR (or if negative). If cultures grow other organism, select other, more targeted agents to complete the course of treatment. If cultures are negative, then select other, more targeted agent(s) for most scenarios.<br><br>Use: Appropriate for SSI caused by a highly resistant GNR. May be appropriate if SSI following GI, GU, or GYN surgery but cultures remain negative. Guidelines suggest cephalosporin + metronidazole in this scenario, however.[5]

© 2019 Anderson DJ et al. *JAMA Network Open*.

	If no cultures were obtained, provide education about importance of culture data to guide antimicrobial therapy. Push for microbiologic diagnosis whenever possible (e.g. drain abscesses to get micro data).<br><br>**Duration**: Depends on extent of SSI<br><br>• Superficial SSI - 5-10 days<br>• More invasive SSI - **Do not provide duration recommendation** (duration depends on need for I&D, type of procedure, implant)
**SKIN AND SOFT TISSUE INFECTION (SSTI)[5]**	**Avoid**: Not appropriate if cultures negative for highly resistant GNR.<br><br>**Use:** Acceptable to continue if no cultures but skin infection had signs of deeper infection (bullae, skin sloughing), severe sepsis, or concern for necrotizing fasciitis. Appropriate if non-purulent cellulitis coupled with a) severe infection (ICU or hypotensive), b) immunocompromised, or c) worsening or no improvement in clinical syndromes.<br><br>***Note that erythema of skin does not regress quickly, even with appropriate therapy***<br><br>**Duration:** 5 days for uncomplicated SSTI. May require 2 or more weeks if multiple I&Ds required (signs of severe sepsis/septic shock) OR immunocompromised.
**DIABETIC FOOT INFECTIONS (DFI)[6]**	***Note: Many diabetic foot ulcers are NOT infected. Ask prescriber if there was purulent drainage OR evidence of at least 2 signs of infection (erythema, warmth, tenderness, pain, or induration) at the time of diagnosis.***<br><br>**Avoid:** If none of above, not appropriate; only local wound care and off-loading is needed. Not appropriate if cultures fail to grow highly-resistant GNR. Not appropriate if oral option is reasonable, systemic symptoms resolved, and no deep infection (i.e, osteo).<br><br>**Use:** Appropriate if h/o PA and signs of infection. Appropriate if severe/systemic symptoms are unresolved.<br><br>***Push for culture data, preferably from "good" specimens (ie, OR specimen, deep culture of bone if suspected osteo, or collection of pus; not a simple wound swab). On the other hand, wound swabs that DO NOT grow PA can be used to recommend narrowing therapy from P/T or CP.***<br><br>**Duration:** Depends on type of infection<br><br>• Mild DFI – switch to orals to complete 1-2 weeks<br>• Moderate to severe DFI – 2-3 weeks, though duration depends on need for I&D, bone involvement (confirmed osteomyelitis requires debridement followed by 6 weeks of IV antibiotics), clinical improvement
**BACTEREMIA**	**General**:<br><br>• Determine the primary source of infection to determine best antibiotic and clinical management. ID consultation may be required.<br>• Duration recommendations (in general) will need to be made after the full diagnostic workup is complete.

© 2019 Anderson DJ et al. *JAMA Network Open*.

	o An important factor to consider is if the primary source is potentially polymicrobial (e.g. abdominal or DFI), even if only a single pathogen is identified in the blood.<br>▪ If so, then broader antibiotic coverage for the polymicrobial infection may be clinically required.<br>• The guidelines below are based on the assumption that the primary infectious source includes *only* the identified pathogen in the blood.<br>**Avoid**: Not appropriate if culture fails to grow a highly-resistant GNR. Target therapy to more narrow agents to complete treatment.<br><br>**Use:** May be appropriate if blood culture grows Enterococci in setting of intra-abdominal infection.<br><br>**Duration:** Treatment duration of GNR BSI is too complicated to provide final duration.
**EMPIRIC SEPSIS**	**General:**<br><br>• Determine the primary source of infection to determine best antibiotic and clinical management.<br>o If infection source has been determined, refer to sections for that specific type of infection.<br>• If infection source remains unclear, then determine if the patient has clinically improved on empiric therapy.<br>o Determine if patient has risk factors for PA infection and the working clinical diagnosis or most likely source.<br>***If source not determined,***<br><br>**Avoid:** Not appropriate if no PA RF and clinically improving. May not be appropriate if no PA RF but not clinically improving. If unwilling to stop, suggest plan to slowly remove antibiotics. Alternatively, may suggest they obtain an ID consult if available.<br><br>**Use:** Appropriate if microbiological specimen suggests highly-resistant GNR infection<br><br>**Duration:** Do not provide treatment duration recommendations for sepsis of unclear etiology.
**INTRA-ABDOMINAL INFECTION**[7]	***Guidelines separate recommendations into several different types of intra-abdominal infections (e.g., biliary, diverticulitis, peritonitis, etc).***<br><br>**Avoid:** Inappropriate if cultures obtained and no evidence of highly resistant GNR. Inappropriate if no cultures and not severe infection (i.e., hypotension or ICU). Alternatives such as cefazolin + metronidazole are likely as effective.<br><br>**Use:** Appropriate if cultures grow highly resistant GNR. Appropriate if no cultures and infection is severe or followed a procedure (i.e, healthcare-associated). Though alternatives are likely as effective (e.g., ceftazidime and metronidazole).

© 2019 Anderson DJ et al. *JAMA Network Open*.

eReferences 2:

1. Guidelines for the management of adults with hospital-acquired, ventilator-associated, and healthcare-associated pneumonia. Am J Resp Crit Care Med 2005;171:388-416.

2. Mandell LA, Wunderink RG, Anzueto A, et al. Infectious Diseases Society of America/American Thoracic Society consensus guidelines on the management of community-acquired pneumonia in adults. Clin Infect Dis 2007;44 Suppl 2:S27-72.

3. Gupta K. et al. International Clinical Practice Guidelines for the Treatment of Acute Uncomplicated Cystitis and Pyelonephritis in Women: A 2010 Update by the Infectious Diseases Society of America and the European Society for Microbiology and Infectious Diseases. Clin Infect Dis 2011;52(5):e103–e12.

4. Nicolle LE et al. Infectious Diseases Society of America Guidelines for the Diagnosis and Treatment of Asymptomatic Bacteriuria in Adults. Clin Infect Dis 2005; 40: 643-54.

5. Stevens DL et al. Practice Guidelines for the Diagnosis and Management of Skin and Soft Tissue Infections: 2014 Update by the Infectious Diseases Society of America. Clin Infect Dis 2014; doi: 10.1093/cid/ciu296.

6. Lipsky BA et al. 2012 Infectious Diseases Society of America Clinical Practice Guideline for the Diagnosis and Treatment of Diabetic Foot Infections. Clin Infect Dis 2012;54(12):132-173.

7. Solomkin JS et al. Diagnosis and management of complicated intra-abdominal infection in adults and children: guidelines by the Surgical Infection Society and the Infectious Diseases Society of America. *Clin Infect Dis.* 2010;50(2):133-64.

© 2019 Anderson DJ et al. *JAMA Network Open*.
