# Supplementary material for: Feasibility of Core Antimicrobial Stewardship Interventions in Community Hospitals
Source: JAMA Netw Open. 2019 Aug 16;2(8):e199369. doi: 10.1001/jamanetworkopen.2019.9369 (PMC6704742; doi:10.1001/jamanetworkopen.2019.9369)
Supplement: Supplement 2. — Trial Protocol [file jamanetwopen-2-e199369-s002.pdf]

Antibacterial Resistance Leadership Group (ARLG)

A Multicenter, Three-Stage Cluster-Randomized Historically Controlled Crossover Trial to Determine the Feasibility and Outcomes from Two Antimicrobial Stewardship Interventions in Community Hospitals

Funding Sponsor:

National Institute of Allergy and Infectious Diseases (NIAID)

Funding Mechanism: 1UM1AI104681-01

Protocol Date:	May 07, 2014
Protocol Version:	1.0

Principal Investigator:

Deverick Anderson, MD, MPH

Professor of Medicine – Infectious Diseases

Duke University Medical Center

Duke Box 102359

Hanes House, Room 165
Durham, NC 27710

Telephone: 919-684-4596

Fax: 919-681-7494

E-mail: deverick.anderson@duke.edu

18

STATEMENT OF COMPLIANCE

This study will be conducted in compliance with the applicable principles and regulatory requirements from the United States Code of Federal Regulations (CFR), including 21 CFR 56 (institutional review board [IRB]) and 21 CFR 50 (informed consent) and to the principles outlined in applicable ICH guidelines.

STUDY PRINCIPAL INVESTIGATOR SIGNATURE

The signature below documents the review and approval of this protocol and provides the necessary assurances that this study will be conducted according to all stipulations of the protocol, including all statements regarding confidentiality, and according to local legal and regulatory requirements and to the principles outlined in applicable U.S. federal regulations and ICH guidelines.

Deverick Anderson, MD, MPH

ARLG Study Principal Investigator

Signature

Date

TABLE OF CONTENTS

Statement of Compliance and Signature Page i
Table of Contents ii
List of Abbreviations iv
Protocol Synopsis v
Schematic/Description of Study Design vi

1 Key Roles 8
2 Background Information and Scientific Rationale 9
2.1 Background Information 9
2.2 Scientific Rationale 9
2.3 Potential Risks and Benefits 9
2.3.1 Potential Risks 9
2.3.2 Benefits 9
3 Objectives 10
3.1 Study Hypotheses and Objectives 10
3.1.1 Hypotheses 10
3.1.2 Primary Objectives 10
3.1.3 Secondary Objectives 11
3.1.4 Exploratory Objective 11
4 Study Design 12
4.1 Study Population 12
4.1.1 Selection of the Study Population 12
4.1.2 Inclusion/Exclusion Criteria 12
4.1.3 Treatment Assignment Procedures 13
4.1.4 Stewardship Strategy Descriptions 13
4.1.5 Termination of Study 14
5 Study procedures 15
5.1 Data Collection 15
5.2 Other Study Procedures 16
6 Study Product Description 17

6.1 Concomitant Medications/Treatments 17
7 Assessment of Safety 18
8 Clinical Monitoring 19
9 Statistical Considerations 20
9.1 Design and Sample Size Considerations 20
9.2 Planned Interim Analyses 21
9.3 Participant Enrollment and Follow-Up 21
9.4 Analysis Plan 22
9.4.1 Analysis 22
9.4.2 Data Acquisition 24
9.4.3 Limitations and Potential Solutions. 25
10 Study Implications 26
11 Participant Confidentiality 27
12 Informed Consent Process 28
13 Source Documents and Access to Source Data/Documents 29
14 Quality Control and Quality Assurance 30
15 Ethics/Protection of Human Participants 31
15.1 Institutional Review Board 31
15.2 Informed Consent 31
15.3 Data Confidentiality 31
15.4 Study Discontinuation 31
16 Data Handling and Record Keeping 32
16.1 Data Management Responsibilities 32
16.2 Data Capture Methods. 32
16.3 Study Data Retention 34
16.4 Protocol Deviations 34
17 Publication Policy 35
18 References 36

LIST OF ABBREVIATIONS

AB	antibiotic
AE	adverse event
AS	antibiotic stewardship
CAP	community-acquired pneumonia
DASON	Duke Antimicrobial Stewardship Outreach Network
DCRI	Duke Clinical Research Institute
DICON	Duke Infection Control Outreach Network
DOCR	Duke Office of Clinical Research
DOT	days of therapy
EHR	electronic health record
HCAP	healthcare-associated pneumonia
IDSA	Infectious Diseases Society of America
IRB	institutional review board
MRN	medical record number
PAR	post-antibiotic prescription review
PI	principal investigator
TMP-SMX	trimethoprim-sulfamethoxazole
UTI	urinary tract infection

PROTOCOL SYNOPSIS

Protocol Title:	A Multicenter, Three-Stage Cluster-Randomized Historically Controlled Crossover Trial to Determine the Feasibility and Outcomes from Two Antimicrobial Stewardship Interventions in Community Hospitals
Phase:	Not Applicable
Products:	Not Applicable
Objectives:	To evaluate the feasibility of implementing antimicrobial restriction and preauthorization vs. prospective audit and feedback in resource-limited community hospitals
Study Design:	Three-stage cluster-randomized historically controlled crossover trial
Study Population:	Patients who receive targeted or alternative antimicrobials, hospitalized in one of the four community hospitals
Number of Participants:	Not Applicable
Number of Sites:	4
Duration of Participant Participation:	Participation is at the hospital level. All hospitals will participate for 13 months.
Dose Schedule:	Not Applicable
Estimated Start:	August 1st 2014
Estimated Time to Complete Enrollment:	13 months for study duration

STUDY DESIGN SCHEMA

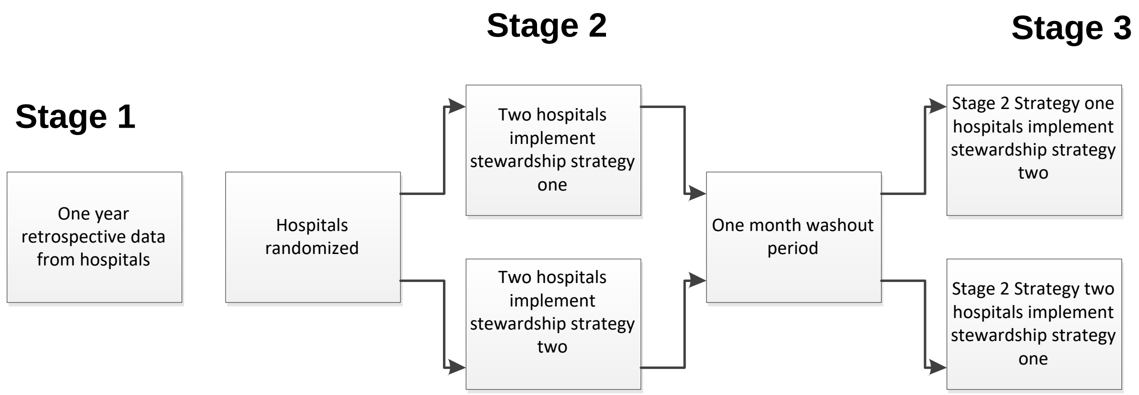

STUDY DESIGN POPULATION

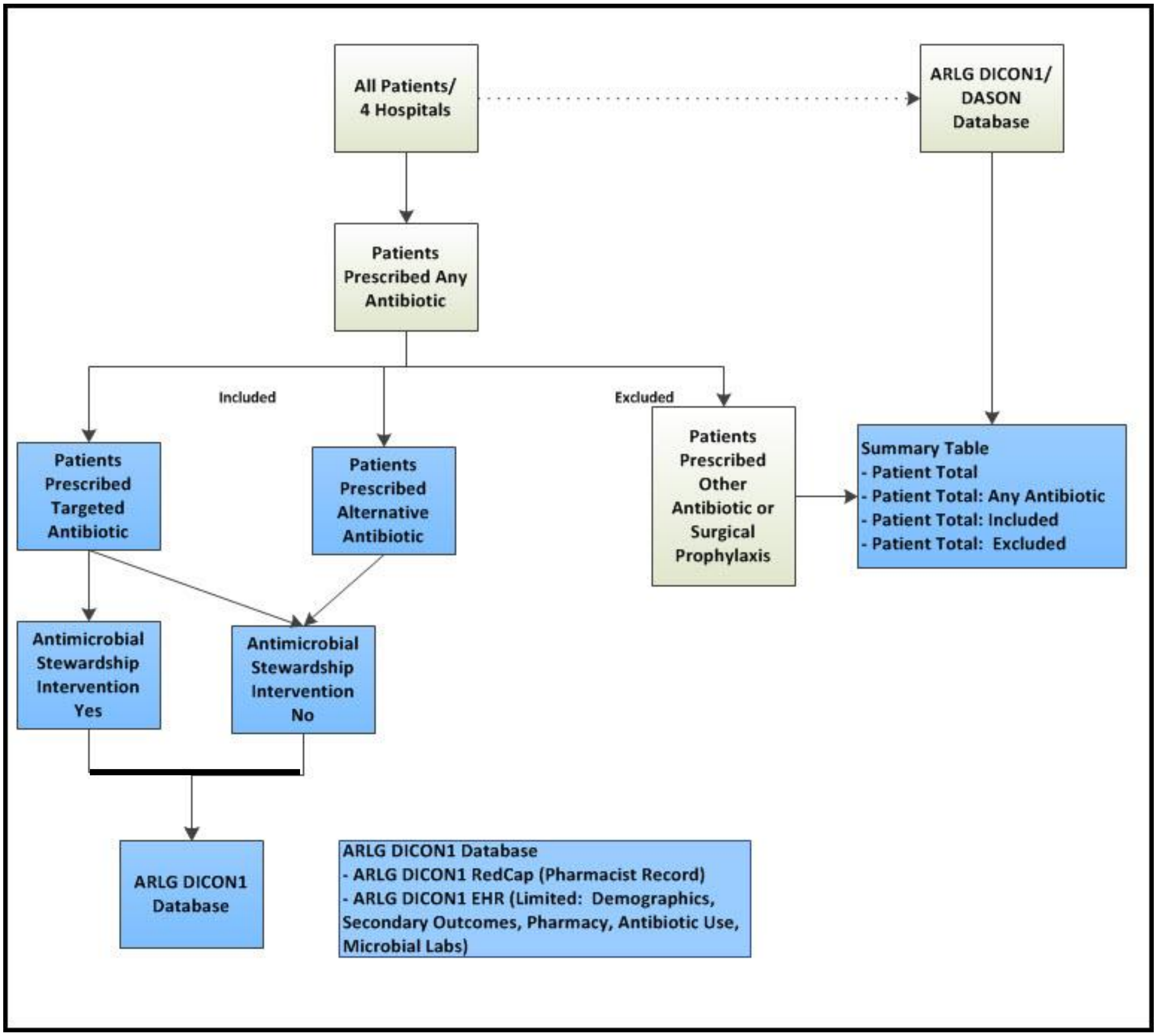

1 KEY ROLES

For questions regarding this protocol, contact:

A) Study Principal Investigator:

Deverick Anderson, MD, MPH
Professor of Medicine – Infectious Diseases
Duke University Medical Center
Duke Box 102359
Hanes House, Room 165
Durham, NC 27710
Telephone: 919-684-4596
Fax: 919-681-7494
E-mail: deverick.anderson@duke.edu

2 BACKGROUND INFORMATION AND SCIENTIFIC RATIONALE

2.1 Background Information

Hospitals in the United States have observed increasing rates of drug-resistant pathogens and healthcare facility associated *Clostridium difficile* infection, leading to significant burdens of morbidity, mortality, and hospital costs.[1-3] A causal relationship between antibiotic exposure and acquired drug resistance is well established. However, 30% to 50% of antibiotic use in US hospitals is inappropriate.[1,4] Hospital-level quality improvement programs to improve antibiotic stewardship (AS) are an essential intervention to curb these concerning trends.[5]

2.2 Scientific Rationale

The Infectious Diseases Society of America (IDSA) guidelines recommend two "core" strategies for AS: 1) antimicrobial restriction and preauthorization and 2) prospective audit with intervention and feedback.1 Community hospitals have limited or no resources and staff dedicated to AS.6 Thus, understanding which of the core strategies is most effective in this practice setting would assist in appropriate allocation of limited resources.

2.3 Potential Risks and Benefits

2.3.1 Potential Risks

Potential risks are minimal due to the fact that all patients will continue to receive treatment per usual care. This study will provide an extra level of examination into the appropriateness of the prescribed antibiotic through AS interaction of prescribing physicians with trained pharmacists.

2.3.2 Benefits

Patients may benefit from having more attention paid to the course of treatment for their infections. Patients may receive more appropriate antimicrobial treatment, including using appropriate antimicrobial agents when an infection is present and stopping antimicrobial agents when they are unnecessary.

3 OBJECTIVES

The purpose of this study is to evaluate the feasibility of implementing antimicrobial restriction and preauthorization vs. post antibiotic prescription review in resource-limited community hospitals.

3.1 Study Hypotheses and Objectives

3.1.1 Hypotheses

- Primary Feasibility Hypothesis

The protocol of two stewardship strategies [Antimicrobial restriction and preauthorization and post-antibiotic prescription review] will receive approval at hospitals. The two stewardship strategies will both be successfully implemented, including training of local pharmacists (PharmDs), at all four hospitals.

Secondary Stewardship Hypotheses

1. Antimicrobial restriction and preauthorization will be less resource-intensive for hospitals (e.g., set-up time, approval time, personnel resources) than post-antibiotic prescription review (PAR) and feedback.
2. Prescribers will consider preauthorization to be intrusive to clinician autonomy and patient care.
3. The cost of performing PAR by a trained pharmacist will be offset by the cost savings achieved by decreasing antimicrobial cost.
4. Both strategies will lead to a decrease in utilization for targeted antimicrobials relative to utilization in the period prior to implementation of the stewardship strategies.
5. Decreases in targeted antimicrobial utilization from antimicrobial restriction will be offset by increases in use of alternative antibiotics.
6. The strategy of antimicrobial restriction and preauthorization will be less effective at reducing overall antimicrobial use than the strategy of prospective audit and feedback, as it will not shorten duration of antimicrobial therapy.

3.1.2 Primary Objectives

Feasibility Objectives

1. To evaluate having the protocol of two stewardship intervention strategies approved by hospital administration and committees
2. To evaluate the training of local PharmDs in administration of the two stewardship strategies

3. To evaluate the initiation and implementation of the two stewardship strategies at four hospitals

3.1.3 Secondary Objectives

To estimate and compare the time required of the pharmacist to administer each strategy

1. To estimate and compare the resource utilization of each strategy, where resource utilization includes pharmacist time and cost of antibiotics.
2. To compare the days of therapy (DOT) for antibiotics of interest between each strategy

   DOT will be measured as the number of days a patient receives each targeted and alternative antibiotic during the hospital admission.

3. To compare patient-specific outcomes between each strategy, including but not limited to the following:
   a. Total hospital days
   b. Death
   c. Intensive care unit admission
4. To compare the prescriber and pharmacist perceptions of the two stewardship strategies
5. To develop integrated measures of benefit: risk for AS (based on days of therapy, hospital days, and patient outcomes such as ICU admission and survival) and compare strategy effects with respect to these measures

3.1.4 Exploratory Objective

To evaluate the interpretations reached from analysis of data based on traditional (DOT/patient-days) outcomes as compared to newer proposed outcomes (defined in Section 3.1.3 Secondary Objectives, number 5).

4 STUDY DESIGN

This study is a three-stage cluster randomized historically controlled crossover trial designed to evaluate the feasibility of the implementation of two AS strategies. Data will be obtained from four community hospitals in three stages. In stage 1, historical data from each hospital will be collected for the year prior to study initiation from all participating hospitals. These data are used as a control representing current practice. In stage 2, the four hospitals will be divided into two pairs based upon bed size. One hospital from each pair will be randomly assigned to a six-month period of antimicrobial restriction and preauthorization (Strategy 1; stage 2) followed by a six-month period of post-antibiotic prescription review (Strategy 2; stage 3). The other two hospitals will undergo Strategy 2 in stage 2 followed by Strategy 1 in stage 3 to help alleviate the concern for seasonal/temporal effects when comparing the strategies to each other. Temporal/seasonal effects are not controlled when comparing to the historical control. A one-month wash out will be performed between each stage. Three antibiotics will be specifically targeted for intervention: anti-pseudomonal carbapenem of choice at the study hospital, vancomycin, and piperacillin-tazobactam. Utilization of alternative antimicrobials, including fluoroquinolones, cephalosporins, and anti-methicillin-resistant Staphylococcus aureus (anti-MRSA) systemic antimicrobials (e.g., nafcillin, daptomycin, linezolid, ceftaroline, clindamycin, and trimethoprim-sulfamethoxazole [TMP-SMX]) will also be collected.

4.1 Study Population

4.1.1 Selection of the Study Population

Four community hospitals in the Duke Infection Control Outreach Network (DICON) will be recruited for this study.[7] Ideally, hospitals will have no teaching affiliation, bed size <300, and no existing antimicrobial formulary restriction and preauthorization or PAR practices in place at study start.

Drug use Identification: All study hospitals will have electronic systems that track antimicrobial prescriptions through orders, electronic medication administration records (eMAR), bar-coded medication administration (BCMA), or dispensing data.

4.1.2 Inclusion/Exclusion Criteria

Inclusion criteria: All adult and pediatric patients admitted to the study hospital who are prescribed targeted or alternative antimicrobial agent(s) will have data collected from their medical records.

Exclusion criteria: Any patient not meeting the criteria above will be excluded. Patients who receive < 24 hours of surgical prophylaxis with a targeted or alternative antimicrobial will be excluded.

4.1.3 Treatment Assignment Procedures

The four hospitals will be divided into two pairs based upon bed size and in discussion with the protocol team. One hospital in each pair will implement Stewardship strategy 1 followed by Stewardship strategy 2. The other hospital in each pair will implement Stewardship strategy 2 followed by Stewardship strategy 1. The order of the strategies will be randomly generated at the SDMC and communicated to the local PharmD and pre-identified hospital staff.

4.1.4 Stewardship Strategy Descriptions

Hospitals will have dedicated clinical pharmacist time for preauthorization or post-antibiotic review for the three targeted antibiotics in each arm of the study. One PharmD at each site will receive standardized training by study personnel in order to address common questions and anticipated arguments, and to establish a robust knowledge base regarding the targeted antimicrobials (anti-pseudomonal carbapenem of choice at the study hospital, vancomycin, and piperacillin-tazobactam). They will also be trained in conflict management.

Study personnel will provide suggested criteria for appropriate use of each targeted drug. Hospital P&T committees will review, edit (if desired), and approve hospital-specific protocols for appropriate use criteria for each study drug. On-site pharmacists will then use the local criteria to determine whether each prescription falls into protocol vs. non-protocol use and then recommend approval/disapproval and/or alternative therapy to the prescriber in real time.

Each participating pharmacist will be thoroughly trained by study staff prior to the study. Pharmacists will be provided with specific clinical pathways for urinary tract infections (UTIs), community-acquired pneumonia (CAP), healthcare-associated pneumonia (HCAP), bacteremia, and “other” uses of targeted antimicrobial. Clinical pathways will be developed jointly by study personnel and based on Centers for Disease Control and Prevention pilot projects. Clinical pharmacists will determine appropriateness of therapy based on study clinical pathways, their baseline knowledge, and acquired knowledge derived by training from study personnel. Time spent performing the two stewardship strategies will be supported by grant funds. The designated pharmacist will be involved in two critical components of the protocol: a) completing the intervention and b) documenting the outcome of the intervention.

<u>Stewardship Strategy 1 – Antimicrobial restriction and Pre-authorization</u>. Following approval of the protocol at study hospitals, all prescriptions for targeted antibiotics will require phone approval by the trained PharmD. Prescribers will be instructed to discuss the patient details and the rationale for the desired antimicrobial with the PharmD. Specifically, prescribers must contact the pharmacist via pager or phone call to discuss the rationale for using the targeted antibiotic. The pharmacist will discuss the rationale and the specific clinical scenario with the prescriber and will decide if the targeted antibiotic is approved or denied. If the pharmacist denies the use of the targeted antibiotic, the pharmacist will provide recommendations for alternative antibiotics for the specific clinical scenario. The pharmacist will then document this interaction in the REDCap database, documenting which targeted antibiotic was requested, the prescriber’s rationale for requesting the targeted antibiotic, the patient’s symptoms and pertinent

clinical data at the time of the request, whether the request met the criteria for use of the targeted antibiotic (or not), and the pharmacist's recommendation. Prescriptions for targeted antibiotics will be eligible for intervention if written between 7am and 6pm Monday through Friday. Off-hours prescriptions will be reviewed for approval for continuation the following morning.

Stewardship Strategy 2 – Post-antibiotic Review and Feedback. Following approval of the protocol at study hospitals, all prescriptions for targeted antibiotics will be reviewed by the study pharmacist approximately 72 hours after initially written. The clinical pharmacists will review a list of patients receiving the targeted antibiotics on a daily basis to identify patients who have received one or more targeted antibiotics for 72 hours (± 24 hours). The pharmacist will review and document the patient's current symptoms, pertinent clinical data, and the indication for the targeted antibiotic documented in the chart. Based on this review, the pharmacist will decide if the targeted antibiotic is necessary and/or if it needs to be modified. If a change is recommended, the pharmacist will then contact the prescriber (i.e., by pager, by phone call, or during clinical rounds) to discuss the pharmacist's recommendations. The interaction will be documented in the REDCap database, including whether or not the prescriber is going to follow the pharmacist's advice. If the pharmacist concludes that the antibiotic is appropriate, then he or she will document that the antibiotic is appropriate but will not be required to contact the prescriber.

Follow up: Strategy 1 and Strategy 2 -- For both strategies, the pharmacist will review the patient's chart approximately five days after interacting with the prescriber to document whether the pharmacist's recommendation was followed. The pharmacist will specifically document if the recommendation was followed, the recommended duration of antibiotic, whether the dose of the antibiotic was appropriate, and the ultimate infection diagnosis. If the patient has already been discharged, the pharmacist will review the patient record to gather this information.

4.1.5 Termination of Study

This study may be terminated at any time by the principal investigator (PI) in consultation with the Antibacterial Resistance Leadership Group (ARLG).

5 STUDY PROCEDURES

5.1 Data Collection

- Data will be collected on the following topics:
- Feasibility
- Strategies
  - PharmD chart and prescription review
  - PharmD/Prescriber communication
  - PharmD follow-up
- Pharmacist time
- Pharmacist and Prescriber perceptions (questionnaires)
- Secondary/Exploratory Objectives: DASON database (Pharmacy data, Admit/Discharge Table, Billing ICD9, Microbiology Lab, Bed Flow)
- The PharmD will provide patient data from two time points: first contact and at the time of discharge. In strategy 1, first contact will be the PharmD/prescriber contact and discussion. In strategy 2, first contact will be the date of the PharmD review (approximately 72 hours after the targeted antibiotic was started).

Endpoints

Primary endpoints (measured at the hospital level):

- Approval of interventions by hospital administration and committees
- Successful completion of training of a local PharmD
- Initiation of the intervention protocols

Secondary endpoints (measured at the patient level):

- DOT for each of the targeted and alternative antibiotics
- Duration of hospitalization
- Admission to the ICU (yes/no)
- Duration in ICU
- Discharged vs. death
- Benefit: risk measures to be developed as composites of other outcomes (details provided in an analysis plan)

Exploratory endpoints (measured at the patient level):

Comparison of utilization outcomes

Data Monitoring

No formal interim analyses involving hypothesis testing are planned. Data from the retrospective portion of the study will be cleaned and transmitted upon completion to conduct further cleaning and prepare for analyses.

Pharmacists will be provided with a data entry guideline (DEG) to ensure consistent data collection across participating hospitals.

5.2 Other Study Procedures

We will not enroll subjects as part of this protocol. Therefore, the following sections are not applicable:

Screening

1. Enrollment/baseline
2. Follow-up
3. Final study visit
4. Follow-up safety phone call
5. Early termination visit
6. Unscheduled visit
7. Laboratory evaluations

6 STUDY PRODUCT DESCRIPTION

Not applicable

6.1 Concomitant Medications/Treatments

Not applicable

7 ASSESSMENT OF SAFETY

We are not enrolling patients; therefore, the following sections are not applicable:

1. Specifications of safety parameters
2. Methods and timing for assessing, recording, and analyzing safety parameters
3. Guidelines for assessing intensity of an adverse event (AE)
4. Guidelines for determining causality
5. Discontinuation due to AEs
6. Reporting procedures (for AE)
7. Type and duration of follow-up of participants after AEs
8. Halting rules
9. Safety oversight

8 CLINICAL MONITORING

ICH E6 states that the purpose of monitoring is to ensure the rights of subjects, obtain accurate data, and conduct the trial in accordance with protocol and applicable regulations. For the purposes of this protocol, a monitoring plan will be developed to ensure that three areas are adequately addressed.

Key areas that will be outlined will be the qualification of hospital personnel to conduct the trial, regulatory requirements (e.g. institutional review board [IRB] review), protocol training, data quality monitoring procedures, hospital data completion expectations (e.g. completeness, frequency), and pathways for issue escalation as well as for resolving any general questions at the hospitals.

9 STATISTICAL CONSIDERATIONS

9.1 Design and Sample Size Considerations

This is a feasibility study to evaluate if four hospitals can implement two antimicrobial stewardship interventions. Analysis of the primary feasibility components will be descriptive (a summary as to whether each hospital was able to successfully implement each of the interventions). No hypothesis testing will be completed for these summaries.

The secondary stewardship analyses may include hypothesis testing and estimation of the difference to the control as well as between the two stewardship strategies.

This study will collect data on all patients prescribed a targeted or alternative antibiotic at each of four hospitals, except patients who receive < 24 hours of surgical prophylaxis. The three targeted antibiotics are:

- Anti-pseudomonal carbapenem of choice at the study hospital
- Vancomycin
- Piperacillin-tazobactam.

The alternative antimicrobials of interest are:

- Fluoroquinolones
- Cephalosporins
- anti-MRSA systemic antimicrobials (e.g., nafcillin, daptomycin, linezolid, ceftaroline, clindamycin, and TMP-SMX).

This study will not target a specific number of patients to enroll. Instead, it will be conducted for specific periods of time. Each hospital will implement its first strategy for six months, followed by an one-month period with no strategy, and then a second, six-month period for the second strategy, for a total duration of 13 months. Additionally, one year of historical data from the four hospitals will be analyzed.

Although this study does not target a specific number of participants, hospitals are expected to admit at least 600 patients per month. Thus, we expect to have at least 3600 patients included in analyses for each six-month intervention period. When conducting within-hospital analyses, a conservative sample size of 3000 patients during each intervention period will achieve 97% power to detect a difference between the group proportions (e.g., of patients with no [0] DOT) of 0.0500 when the proportion in one group is 0.50 using an alpha of 0.05. The power for detecting a difference as a function of sample size is illustrated in Figure *9-1*:

Figure 9-1:

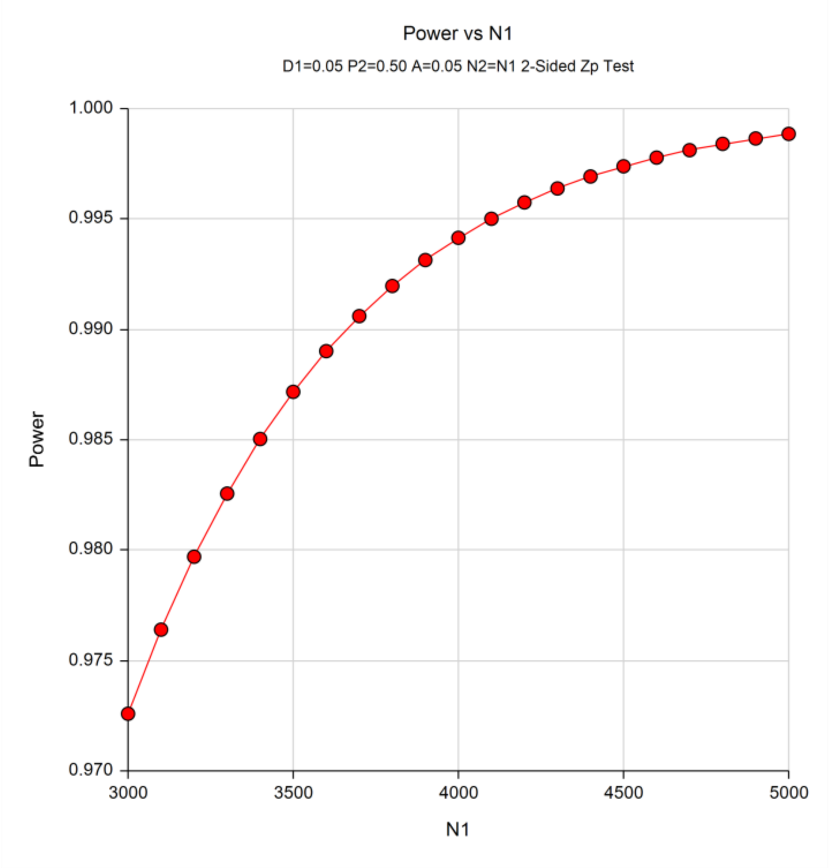

9.2 Planned Interim Analyses

As this is a strategy study that enrolls hospitals, there will be no planned interim analyses for safety in this protocol. We expect to summarize historical data while the prospective portion of the study is ongoing.

9.3 Participant Enrollment and Follow-Up

Data from participants admitted to the four hospitals during each of the two six month strategy periods are entered into the database. The data from admission until death or discharge are collected.

9.4 Analysis Plan

9.4.1 Analysis

9.4.1.1 Primary feasibility summaries

The primary analyses will be descriptive (a summary as to whether each hospital was able to successfully implement each of the interventions). No hypothesis testing will be completed for these analyses.

9.4.1.2 Secondary analyses

The analyses of the secondary objectives will consist of multiple analyses to address the hypotheses. The endpoints required for these analyses include, but are not limited to:

- Days of targeted and alternative antimicrobial use
- Prescriptions for targeted and alternative antimicrobials
- Days in hospital
- Death
- ICU admission
- Survey of provider perceptions
- Costs including cost of pharmacist and antimicrobial costs

9.4.1.3 Discussion of cluster randomized design and the key secondary analysis

This antimicrobial stewardship study will be implemented at four hospitals, where each hospital is considered a single cluster. The four hospitals participating in this study will be paired by size. One hospital in each pair will receive Strategy 1 followed by Strategy 2, while the other hospital in each pair will receive Strategy 2 followed by Strategy 1. The ordering within each pair will be randomly assigned.

Cluster randomized trials can be analyzed at the cluster level or the patient level. Ideally, analyses are conducted on the cluster level as this is the unit of randomization. However, we will analyze this study using the patient as the unit of analyses as there are only four clusters.

Since randomization occurs at the cluster level but analyses must be conducted at the patient level, the benefits of randomization (i.e., expectation of balance of confounding factors) cannot be assumed. The small sample size of 4 clusters may also negate the benefits of randomization. The crossover nature of the design will help to balance confounding factors as each hospital can serve as its own control. However, as the crossover occurs at the cluster level, and the patients are neither randomized nor crossed-over, it does not have the same advantages as a standard crossover trial and it does not eliminate within-patient variation; the analyses cannot be “paired”.

Since the effect of the intervention is not only a function of the intervention itself but also of the pharmacist, there is potential for considerable site variation in response. Thus each of the four sites will be evaluated separately. Stratified pooled analyses may also be conducted.

For each site and each contrast (Intervention 1 vs. control; Intervention 2 vs. control; and Intervention 1 vs. Intervention 2), the difference in outcomes will be estimated using 95% confidence intervals. Examples for binary and continuous outcomes are displayed in Figure 9-2 and Figure 9-3, respectively.

Figure 9-2

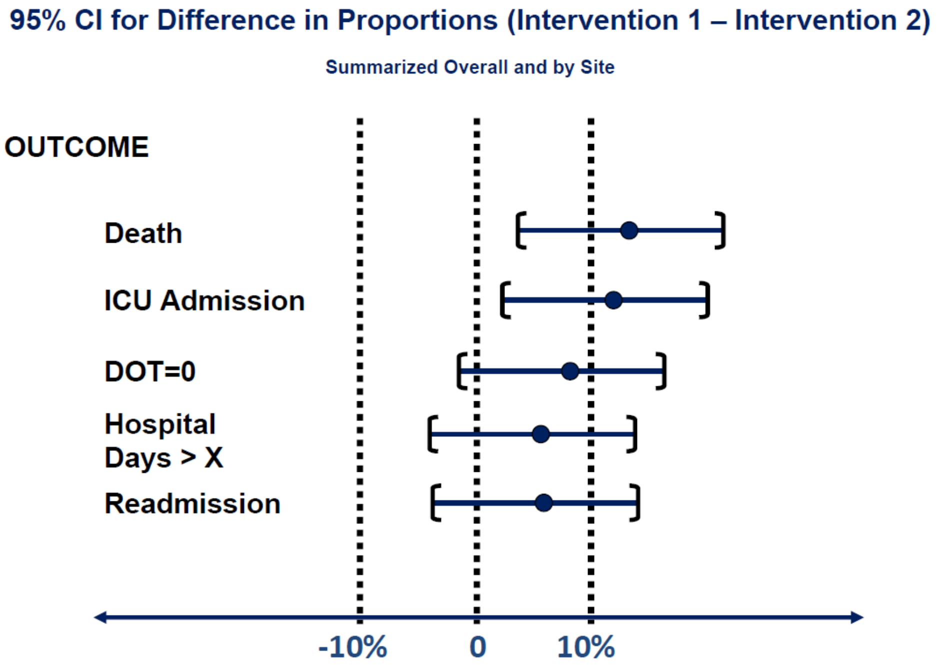

Figure 9-3

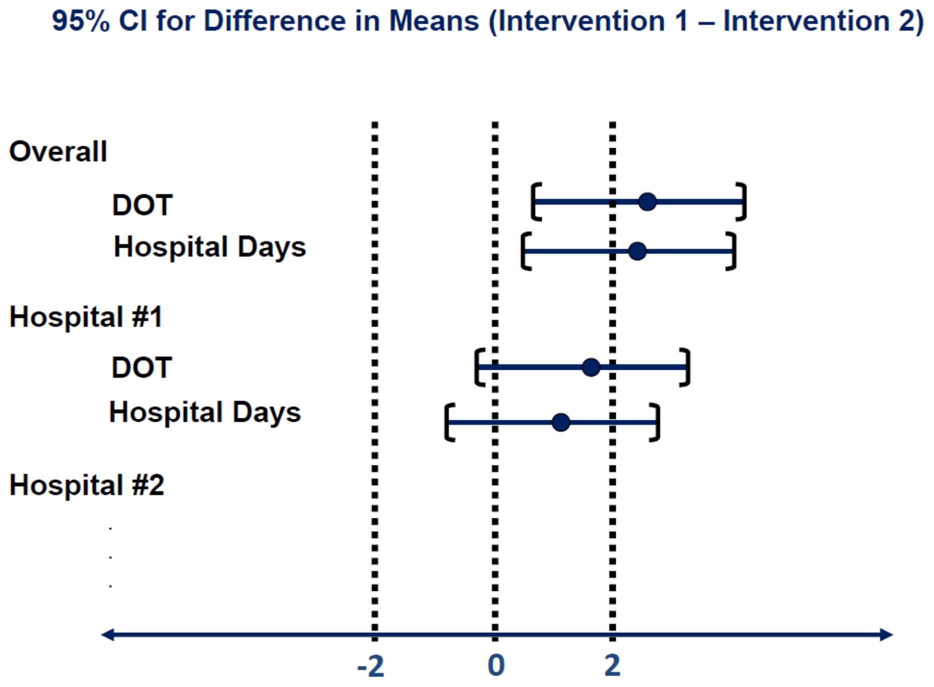

Site heterogeneity of treatment effects will be explored by modeling outcome as a function of treatment, period, site, and period*site interaction. Age, sex, and race will also be included to control for confounding by demographic variables. Treatment and period effects will be fixed. Site and period*site effects will be random.

Exploratory analyses of the overall benefits and risks of the AS interventions will also be conducted using methods that are currently being developed.

9.4.2 Data Acquisition

Cost and feasibility data will be obtained using hospital cost data (antibiotics and pharmacy FTE), questionnaires, and notes/observations created during interactions with hospital personnel. Patient endpoint data will be obtained from electronic pharmacy antibiotic prescribing data (start and stop dates for all antibiotics) and hospital databases (e.g., ADT data for admit/discharge dates for all patients; patient status database (mortality); and diagnosis code databases).

9.4.3 Limitations and Potential Solutions.

The above study approach has limitations. First, the study is essentially non-randomized. Even randomization of hospitals cannot guarantee the balance of confounding factors on the unit of analyses (patients). The cross-over design helps to alleviate this concern as each hospital would be expected to enroll similar populations into each intervention period.

Second, our designed strategies may miss intervention opportunities. Our study design includes pharmacist interventions during business hours on Monday through Friday and does not include weekend interventions. This inclusion criterion will impact Strategy 1 more than Strategy 2, as no alternative approach is available for Strategy 1. To overcome this limitation for Strategy 2, we will allow the intervention to occur within 72 hours of prescription ***± 24 hours***, if necessary. This window for intervention will be adequate to intervene on all antibiotic prescriptions. We believe that this approach is appropriate, as we suspect this approach is likely to mirror how most hospitals will eventually implement each of these interventions; thus, it is more likely to lead to true conclusions regarding the feasibility and outcomes of each of these interventions.

Third, the short study period and limited study sites will hinder our ability to demonstrate definitive changes in our secondary analyses, including changes in antimicrobial utilization. The primary goal of this study, however, is to assess feasibility.

Next, the effectiveness of any stewardship program is likely to vary depending on the experience and expertise of the pharmacist performing the PAR and local hospital culture. In fact, we will specifically measure pharmacist variables (e.g., years of experience, experience with stewardship, and training in infectious diseases) to identify barriers to successful implementation of PAR. We will, however, perform pre-intervention standardized training for all pharmacists by a highly experienced and seasoned pharmacist educator in order to make sure all participating pharmacists have appropriate competency.

Given that the strategies are applied at the hospital level, there is the potential for selective entry, i.e., patients and others may be aware of the stewardship strategy prior to enrolling into the study.

Finally, as the primary control group is historical, subsequent comparisons may be biased by temporal effects. The crossover nature of the trial design helps to alleviate this bias inherent to comparing the AS interventions in pooled analyses.

10 STUDY IMPLICATIONS

Results from this study will provide valuable information for planning a large, multicenter study investigating one or both of the proposed interventions in community hospitals. For example, subsequent research could be limited to one intervention, depending on the feasibility determined in this pilot study. In addition, important parameters will be available to inform power calculations for future cluster-randomized trials.

11 PARTICIPANT CONFIDENTIALITY

Appropriate waivers of consent and HIPAA authorization will be obtained at all sites.

12 INFORMED CONSENT PROCESS

Appropriate waivers of consent and HIPAA authorization will be obtained from all local IRBs overseeing hospitals.

13 SOURCE DOCUMENTS AND ACCESS TO SOURCE DATA/DOCUMENTS

No source documents will be used by this protocol.

14 QUALITY CONTROL AND QUALITY ASSURANCE

The PI will ensure that all study personnel are appropriately trained and applicable documentations are maintained.

The DCRI will implement a Quality Plan to, at a minimum, ensure that activities proposed by the PI to ensure protocol training, data quality and data security are being undertaken.

15 ETHICS/PROTECTION OF HUMAN PARTICIPANTS

15.1 Institutional Review Board

The investigator will ensure that the protocol is reviewed and approved by the appropriate IRB prior to the start of any study activities. The IRB will be appropriately constituted and will perform its functions in accordance with US regulations, ICH Good Clinical Practice guidelines, and local requirements as applicable.

15.2 Informed Consent

This study does not involve the enrollment of participants; appropriate waivers of consent and HIPAA authorization will be obtained from all local IRBs overseeing hospitals.

15.3 Data Confidentiality

The study protocol, documentation, data, and all other information generated by this study will be maintained in a secure manner and will be kept confidential as required by law. In order to meet federal or state regulations, this information may be reviewed by the FDA, IRBs, the sponsor and its representatives, or the NIH.

Data access will be limited to study personnel, and the data will be stored on servers with limited physical access (e.g., locked rooms) and limited electronic access (e.g., password-controlled access to data, computers, and, if applicable, networks). No information concerning the study or the data will be released to any third party without prior written approval of the ARLG.

15.4 Study Discontinuation

This study may be terminated at any time by the PI in consultation with the ARLG.

16 DATA HANDLING AND RECORD KEEPING

16.1 Data Management Responsibilities

The hospitals will enter data into the ARLG DICON1 database via Research Electronic Data Capture (REDCap; see Data Capture Methods below). Other data will come from the ARLG DICON1 EHR database. DICON Network personnel will reconcile the data, strip out the MRN identifiers, and provide DCRI with an ARLG DICON1 limited database with the DICON ID as the subject identifier. These limited datasets will be sent to DCRI as the data coordinating center. DCRI will send the data to the Harvard Statistical Center for analysis.

16.2 Data Capture Methods

REDCap is a toolset and workflow methodology for electronic collection and management of research and clinical trial data. Both REDCap and REDCap Survey systems provide secure, web-based applications that are flexible enough to be used for a variety of types of research, provide an intuitive interface for users to enter data and have real time validation rules (with automated data type and range checks) at the time of entry. These systems offer easy data manipulation with audit trails and reporting for reporting, monitoring and querying patient records, and an automated export mechanism to common statistical packages (SPSS, SAS, Stata, R/S-Plus).

The REDCap program will serve as the portal for data entry at remote study hospital locations. Data entered into this database will be password protected and only accessible by study personnel. All access to this secure separate database will be monitored and logged.

This REDCap database will be created and managed by the DCRI and hosted by DOCR at Duke. MRN will be stripped from REDCap data sent to DCRI.

EHR data from the hospitals are sent to Duke DASON from each of the study hospitals through routine DASON activities. These data files are sent via secure File Transfer Protocol (sFTP) to servers and are available on shared drives kept behind DUHS firewalls. By standard DASON procedure, these data are typically stripped of PHI except dates prior to transmission to DUHS. Individual patients are instead identified by a “DICON ID”. For the purposes of this study, however, we will eliminate the step that removes PHI so that MRN is provided in these files. This specific data variable is required to connect the data received from the pharmacist intervention database to the EHR database. The MRN will be stripped out of the datasets sent to DCRI.

Specific Data Management. Given the number of hospitals involved in the study and the various types and sources of data to be used, consideration must be given to each type and location of data required for the study.

1. Pharmacist intervention forms - These REDCap forms will be created and managed by DCRI and hosted by DOCR at Duke. The pharmacist at each site will complete these forms for each intervention. These data will include PHI such as MRN. Data from these forms represents the only subject level data specifically generated for this study. MRN data will not be included in the transfers to DCRI.
2. Pharmacist time forms - These REDCap forms will be completed by the pharmacist at each hospital and will provide information concerning the time spent on the interventions.
3. Hospital level forms - These REDCap forms will be completed by DASON personnel. These data will include information regarding the approval and implementation of the stewardship strategies at each hospital
4. Questionnaires - These REDCap questionnaires will be completed by the pharmacists and prescribers at each hospital after each strategy is completed (at the end of the six month period). Pharmacists and prescribers will be sent a link to the questionnaires in order to complete. These will not require a REDCap account.
5. Pharmacy data - Antimicrobial utilization data are sent to Duke DASON from each of the study hospitals through routine DASON activities. These data will include PHI such as MRN and dates. These data are created through routine hospital pharmacy activities. These data files will include PHI such as MRN and dates. These data will be transmitted to DUHS via the same sFTP established for routine transmission of data described above. MRN data will not be included in the transfers to DCRI.
6. Bed Flow and Admission-Discharge-Transfer (ADT) data – We will receive bed flow (patient movement within the hospital) and ADT files from study hospitals on a monthly basis through standard DASON activities. These data files will include PHI such as MRN and dates. These data will be transmitted to DUHS via the same sFTP established for routine transmission of data described above. MRN data will not be included in the transfers to DCRI.
7. Demographics data - We will receive data from study hospitals on a monthly basis through standard DASON activities. These data files will include PHI such as MRN and dates. These data will be transmitted to DUHS via the same sFTP established for routine transmission of data described above. MRN data will not be included in the transfers to DCRI.
8. Billing/ICD-9 data - We will receive data from study hospitals on a monthly basis through standard DASON activities. These data files will include PHI such as MRN and dates. These data will be transmitted to DUHS via the same sFTP established for routine transmission of data described above. MRN data will not be included in the transfers to DCRI.
9. Microbiology data - We will receive data from study hospitals on a monthly basis through standard DASON activities. These data files will include PHI such as MRN and dates. These data will be transmitted to DUHS via the same sFTP established

for routine transmission of data described above. MRN data will not be included in the transfers to DCRI.

16.3 Study Data Retention

Research records and data must be kept for a minimum of 6 years after final reporting or publication.

16.4 Protocol Deviations

Not applicable.

17 PUBLICATION POLICY

Following completion of the study, the investigator may publish the results of this research in a scientific journal under the oversight of the Publication Committee of the ARLG.

18 REFERENCES

1. Dellit TH, Owens RC, McGowan JE, Jr., et al. Infectious Diseases Society of America and the Society for Healthcare Epidemiology of America guidelines for developing an institutional program to enhance antimicrobial stewardship. Clinical infectious diseases : an official publication of the Infectious Diseases Society of America 2007;44:159-77.
2. Miller BA, Chen LF, Sexton DJ, Anderson DJ. Comparison of the burdens of hospital-onset, healthcare facility-associated Clostridium difficile Infection and of healthcare-associated infection due to methicillin-resistant Staphylococcus aureus in community hospitals. Infect Control Hosp Epidemiol 2011;32:387-90.
3. Sievert DM, Ricks P, Edwards JR, et al. Antimicrobial-resistant pathogens associated with healthcare-associated infections: summary of data reported to the National Healthcare Safety Network at the Centers for Disease Control and Prevention, 2009-2010. Infection control and hospital epidemiology : the official journal of the Society of Hospital Epidemiologists of America 2013;34:1-14.
4. Moehring RW, Anderson DJ. Antimicrobial Stewardship as Part of the Infection Prevention Effort. Current infectious disease reports 2012;14:592-600.
5. Policy Statement on Antimicrobial Stewardship by the Society for Healthcare Epidemiology of America (SHEA), the Infectious Diseases Society of America (IDSA), and the Pediatric Infectious Diseases Society (PIDS). Infection control and hospital epidemiology : the official journal of the Society of Hospital Epidemiologists of America 2012;33:322-7.
6. Ohl CA, Dodds Ashley ES. Antimicrobial stewardship programs in community hospitals: the evidence base and case studies. Clinical infectious diseases : an official publication of the Infectious Diseases Society of America 2011;53 Suppl 1:S23-8; quiz S9-30.
7. Huenger F, Schmachtenberg A, Haefner H, et al. Evaluation of postdischarge surveillance of surgical site infections after total hip and knee arthroplasty. Am J Infect Control 2005;33:455-62.

Antibacterial Resistance Leadership Group (ARLG) - DICON

A Multicenter, Three-Stage Cluster-Randomized Historically Controlled Crossover Trial to Determine the Feasibility and Outcomes from Two Antimicrobial Stewardship Interventions in Community Hospitals

Statistical Analysis Plan

April 2016

Statisticians:
Lauren Komarow
Matt Finnemeyer

Table of Contents:

1. Introduction 3
2. Core Manuscript writing team 3
3. Study overview 3
3.1 Study Design 3
3.2 Randomization 4
3.3 Hypotheses 5
3.4 Study Objectives 5
3.3.1 Primary objectives and outcomes 5
3.3.2 Secondary Objectives and Outcomes 5
3.3.3 Exploratory objective 6
3.5 General statistical considerations 6
3.5.1 Cluster Randomization and Analysis 6
3.5.2 Study Limitations 7
4. Feasibility Analyses 8
5. Pharmacist Time (Resource utilization) 8
6. Hospital Admissions 8
7. Interventions recorded/tracking of admissions with interventions 9
8. Selected patient characteristics 9
9. REDCap Intervention summary tables 10
10. Days Of Therapy 12
11. Prescriber and Pharmacist perceptions of the two stewardship strategies (Secondary objective 4) 13
12. Resource utilization (cost of antibiotics) 13
13. Patient specific outcomes between each strategy 13

1. Introduction

The purpose of this document is to describe the analyses proposed for the analysis of DICON. The plan includes the key analyses that will form the core of any presentation or publication used to disseminate the primary conclusions of the study.

We intend to do multiple reports for DICON. The potential reports include:

- Feasibility, Provider perceptions, REDCap Summary tables
- Secondary objectives, focusing on Days of Therapy (DOT) and patient specific outcomes including total hospital days, Death and Intensive Care unit admission
- Exploratory objectives.

2. Core Manuscript writing team

To be determined.

3. Study overview

3.1 Study Design

DICON is a three stage, multicenter cluster randomized historically controlled crossover trial to determine the feasibility and outcomes from two antimicrobial Stewardship interventions in community hospitals.

The three stages of the study are:

- Stage 1: One year of retrospective data from hospitals
- Stage 2: Two hospitals implement stewardship strategy one for six months, while the other two hospitals implement stewardship strategy two for six months. This is followed by a one month washout period.
- Stage 3: Strategy 1 hospitals implement strategy 2 and strategy 2 hospitals implement strategy 1.

The two study strategies are:

<u>Stewardship Strategy 1 – Antimicrobial restriction and Pre-authorization</u>. Following approval of the protocol at study hospitals, all prescriptions for targeted antibiotics will require phone approval by the trained PharmD. Prescribers will be instructed to discuss the patient details and the rationale for the desired antimicrobial with the PharmD. Specifically, prescribers must contact the pharmacist via pager or phone call to discuss the rationale for using the targeted antibiotic. The pharmacist will discuss the rationale and the specific clinical scenario with the prescriber and will decide if the targeted antibiotic is approved or denied. If the pharmacist denies the use of the targeted antibiotic, the pharmacist will provide recommendations for alternative antibiotics for the specific clinical scenario. The pharmacist will then document this interaction in the REDCap database, documenting

which targeted antibiotic was requested, the prescriber's rationale for requesting the targeted antibiotic, the patient's symptoms and pertinent clinical data at the time of the request, whether the request met the criteria for use of the targeted antibiotic (or not), and the pharmacist's recommendation. Prescriptions for targeted antibiotics will be eligible for intervention if written between 7am and 6pm Monday through Friday. Off-hours prescriptions will be reviewed for approval for continuation the following morning.

<u>Stewardship Strategy 2 – Post-antibiotic Review and Feedback</u>. Following approval of the protocol at study hospitals, all prescriptions for targeted antibiotics will be reviewed by the study pharmacist approximately 72 hours after initially written. The clinical pharmacists will review a list of patients receiving the targeted antibiotics on a daily basis to identify patients who have received one or more targeted antibiotics for 72 hours (± 24 hours). The pharmacist will review and document the patient's current symptoms, pertinent clinical data, and the indication for the targeted antibiotic documented in the chart. Based on this review, the pharmacist will decide if the targeted antibiotic is necessary and/or if it needs to be modified. If a change is recommended, the pharmacist will then contact the prescriber (i.e., by pager, by phone call, or during clinical rounds) to discuss the pharmacist's recommendations. The interaction will be documented in the REDCap database, including whether or not the prescriber is going to follow the pharmacist's advice. If the pharmacist concludes that the antibiotic is appropriate, then he or she will document that the antibiotic is appropriate but will not be required to contact the prescriber.

<u>Follow up: Strategy 1 and Strategy 2</u> -- For both strategies, the pharmacist will review the patient's chart approximately five days after interacting with the prescriber to document whether the pharmacist's recommendation was followed. The pharmacist will specifically document if the recommendation was followed, the recommended duration of antibiotic, whether the dose of the antibiotic was appropriate, and the ultimate infection diagnosis. If the patient has already been discharged, the pharmacist will review the patient record to gather this information.

3.2 Randomization

The study was randomized by pairs of hospitals based on bed size. The four hospitals participating in the study were paired based on bed size as listed in the AHA Guide, 2013 edition.
The pairs were Pair 1: Maria Parham Medical Center (102 beds) and Wilson Medical Center(220 beds) and Pair 2: Wayne Memorial Hospital (274 beds) and Southeastern Regional Medical Center (373 beds).

The 2 Stewardship strategies were ordered as Option 0: Formulary restriction and Pre-Authorization for the first six months followed by Post-antibiotic prescription review (PAR) for the second six month period or Option 1: PAR for the first six months followed by formulary restriction for the 2nd six months. A binomial outcome was randomly generated (Bernoulli 0.5) in SAS 9.1 for the ordering of the strategies for the smaller hospital in each pair, with the 2nd hospital receiving the strategies in the opposite order. A set seed was used for reproducible results.

The results of the randomization were:

Maria Parham	1. Formulary Restriction/Pre-Authorization	2. PAR
Wilson	1. PAR	2. Formulary Restriction/Pre-Authorization
Wayne	1. PAR	2. Formulary Restriction/Pre-Authorization

Southeastern 1. Formulary Restriction/Pre-Authorization 2. PAR

3.3 Hypotheses

Primary Feasibility Hypothesis

The protocol of two stewardship strategies [Antimicrobial restriction and preauthorization and post-antibiotic prescription review] will receive approval at hospitals. The two stewardship strategies will both be successfully implemented, including training of local pharmacists (PharmDs), at all four hospitals.

Secondary Stewardship Hypotheses

1. Antimicrobial restriction and preauthorization will be less resource-intensive for hospitals (e.g., set-up time, approval time, personnel resources) than post-antibiotic prescription review (PAR) and feedback.
2. Prescribers will consider preauthorization to be intrusive to clinician autonomy and patient care.
3. The cost of performing PAR by a trained pharmacist will be offset by the cost savings achieved by decreasing antimicrobial cost.
4. Both strategies will lead to a decrease in utilization for targeted antimicrobials relative to utilization in the period prior to implementation of the stewardship strategies.
5. Decreases in targeted antimicrobial utilization from antimicrobial restriction will be offset by increases in use of alternative antibiotics.
6. The strategy of antimicrobial restriction and preauthorization will be less effective at reducing overall antimicrobial use than the strategy of prospective audit and feedback, as it will not shorten duration of antimicrobial therapy.

3.4 Study Objectives

3.3.1 Primary objectives and outcomes

Feasibility Objectives

1. To evaluate having the protocol of two stewardship intervention strategies approved by hospital administration and committees
2. To evaluate the training of local PharmDs in administration of the two stewardship strategies
3. To evaluate the initiation and implementation of the two stewardship strategies at four hospitals

3.3.2 Secondary Objectives and Outcomes

To estimate and compare the time required of the pharmacist to administer each strategy

1. To estimate and compare the resource utilization of each strategy, where resource utilization includes pharmacist time and cost of antibiotics.
2. To compare the days of therapy (DOT) for antibiotics of interest between each strategy

DOT will be measured as the number of days a patient receives each targeted and alternative antibiotic during the hospital admission.

3. To compare patient-specific outcomes between each strategy, including but not limited to the following:
   a. Total hospital days
   b. Intensive care unit admission
4. To compare the prescriber and pharmacist perceptions of the two stewardship strategies
5. To develop integrated measures of benefit: risk for AS (based on days of therapy, hospital days, and patient outcomes such as ICU admission and survival) and compare strategy effects with respect to these measures

3.3.3 Exploratory objective

To evaluate the interpretations reached from analysis of data based on traditional (DOT/patient-days) outcomes as compared to newer proposed outcomes (defined in Section 3.1.3 Secondary Objectives, number 5).

3.5 General statistical considerations

- Tables will be presented by Hospital and intervention; each of the 4 sites will be evaluated separately.
  - Stratified pooled analyses may also be conducted
- Dates will not be shown, but converted to days from intervention (intervention) or first hospital contact (feasibility).
- Each Table/Figure/Listing will be annotated with the name and location of the program used to create it, including its location directory.
- All programs that create permanent study-specific derived datasets or that contribute to analyses of the primary or secondary objectives will be validated according to CBAR SOPs
- Categorical variables will be summarized with n and proportion
- Continuous variables will be summarized with mean, sd, median, Q1, Q3, min and max.
- For secondary analyses, there are 3 contrasts of interest
  - PAR vs. historical control
  - Formulary Restriction/Preauthorization (Res/PreA) vs historical control
  - PAR vs Res/PreA
- For each contrast, the difference in outcomes will be estimated using 95% confidence intervals
  - Difference in proportion with 95% CIs for binary outcomes
  - Difference in means with 95% CIs for continuous outcomes

3.5.1 Cluster Randomization and Analysis

This antimicrobial stewardship study was implemented at four hospitals, where each hospital is considered a single cluster. Cluster randomized trials can be analyzed at the cluster level or the patient level. Ideally, analyses are conducted on the cluster level as this is the unit of randomization. However, we will analyze this study using the patient as the unit of analyses as there are only four clusters.

Since randomization occurs at the cluster level but analyses must be conducted at the patient level, the benefits of randomization (i.e., expectation of balance of confounding factors) cannot be assumed. The small sample size of 4 clusters may also negate the benefits of randomization. The crossover nature of the design will help to balance confounding factors as each hospital can serve as its own control. However, as the crossover occurs at the cluster level, and the patients are neither randomized nor crossed-over, it does not have the same advantages as a standard crossover trial and it does not eliminate within-patient variation; the analyses cannot be “paired”.

Since the effect of the intervention is not only a function of the intervention itself but also of the pharmacist, there is potential for considerable site variation in response. Thus each of the four sites will be evaluated separately. Stratified pooled analyses may also be conducted.

3.5.2 Study Limitations

Drug Shortages

A known limitation of this study is that there were drug shortages among the antibiotics of interest while the study was ongoing. This means that differences observed between the two interventions, or the lack of differences observed may be related to drug availability and not the intervention.

Study Design

The study design has limitations. First, the study is essentially non-randomized. Even randomization of hospitals cannot guarantee the balance of confounding factors on the unit of analyses (patients). The cross-over design helps to alleviate this concern as each hospital would be expected to enroll similar populations into each intervention period.

Second, our designed strategies may miss intervention opportunities. Our study design includes pharmacist interventions during business hours on Monday through Friday and does not include weekend interventions. This inclusion criterion will impact Strategy 1 more than Strategy 2, as no alternative approach is available for Strategy 1. To overcome this limitation for Strategy 2, we will allow the intervention to occur within 72 hours of prescription ± 24 hours, if necessary. This window for intervention will be adequate to intervene on all antibiotic prescriptions. We believe that this approach is appropriate, as we suspect this approach is likely to mirror how most hospitals will eventually implement each of these interventions; thus, it is more likely to lead to true conclusions regarding the feasibility and outcomes of each of these interventions.

Third, the short study period and limited study sites will hinder our ability to demonstrate definitive changes in our secondary analyses, including changes in antimicrobial utilization. The primary goal of this study, however, is to assess feasibility. An additional limitation to the short study period, is that there may be temporal effect that will differ across the two study periods

Next, the effectiveness of any stewardship program is likely to vary depending on the experience and expertise of the pharmacist performing the PAR and local hospital culture.
As the primary control group is historical, subsequent comparisons may be biased by temporal effects. The crossover nature of the trial design helps to alleviate this bias inherent to comparing the AS interventions in pooled analyses.

Lastly, DOT is limited to the duration of hospitalization, which ends upon discharge or death.

4. Feasibility Analyses

Purpose: to address the primary feasibility objectives

Summary of REDCap data - Listings by hospital

Approval Information

- Days from Date of initial discussion to date intervention approved
- Met with Pharmacy leadership (y/n)
- Days from initial discussion to Pharmacy Leadership Meeting
- Met with PT committee (y/n)
- Days from initial discussion to PT committee meeting
- Met with hospital administration (y/n)
- Days from initial discussion to Hospital Administration Meeting
- Met with medical executive committee (y/n)
- Days from initial discussion to Medical Executive Meetings
- Met with other (specify) (y/n)
- Days from initial discussion to Other meeting

Hospital Size

- Hospital bed size
- Hospital average patient days (per year) (confirm that this is per year and not per month)

IT Set -Up Time

- IT time required to set up lists

Pharmacist Training, by Hospital

- Number of Pharmacists trained
- Mean Pharmacist training time (hours)

Treatment Assignment – Study period for each Stewardship Strategy, by hospital

- PAR assignments
- Restriction/Pre-Authorization assignments

5. Pharmacist Time (Resource utilization)

Purpose: to summarize pharmacist time by hospital and stewardship strategy. Addresses secondary objective 1, resource utilization of each strategy where resource utilization includes pharmacist time.

- Listing of hours per week by hospital for study period 1 and study period 2.
- Summary, by hospital, of hours per week for period 1 and period 2

6. Hospital Admissions

Purpose: To summarize hospital admissions over the time periods of interest.

Hospital Admissions Tables, by hospital

a. Number of unique admissions by hospital
    1. In the year preceding the start of the study period 1 (October 6, 2013 through October 5, 2014)
    2. Study period 1 (October 6, 2014 through April 3, 2014)
    3. Study period 2 (May 4, 2015 through October 30, 2014)
b. Number of admissions by hospital that received an "Included antibiotic" or "other antibiotic"
    1. In the year preceding the start of the study period 1 (October 6, 2013 through October 5, 2014)
    2. Study period 1 (October 6, 2014 through April 3, 2014)
    3. Study period 2 (May 4, 2015 through October 30, 2014)
c. Number of admissions by hospital that received an "Included antibiotic"
    1. In the year preceding the start of the study period 1 (October 6, 2013 through October 5, 2014)
    2. Study period 1 (October 6, 2014 through April 3, 2014)
    3. Study period 2 (May 4, 2015 through October 30, 2014)

7. Interventions recorded/tracking of admissions with interventions

Example content:

Purpose: to document the number of interventions performed and the number of unique patients per period/strategy/hospital. To document mis-matches across datasets.

Interventions tables

a. Number of stewardship interventions recorded in REDCap
b. Number of unique patients
c. Number of interventions with intervention follow-up information
d. Number of interventions which match to EHR demographics and antibiotics data
e. Number of interventions which match to EHR height/weight information

Number of intervention follow-ups recorded that do not match to an Intervention

Number of interventions per patient per study period, by Hospital

8. Selected patient characteristics

Purpose: to describe the hospital/study population

By Hospital, for the following groups/time periods

a. Year prior to study
b. Period 1 Admission with Included antibiotic, no intervention
c. Period 1 Admission with stewardship intervention
d. Period 2 admission with Included antibiotic, no intervention
e. Period 2 admission with stewardship intervention

- Age/year of birth
- Race
- Sex
- Ethnicity
- Height (cm)
- Weight (kg)
- BMI

9. REDCap Intervention summary tables

Purpose: to summarize the intervention data

a. For categorical variables: Tables showing categorized N and % within Hospital/Study period
b. Table summaries include mean, SD, min, 25th, median, 75th, and max

Intervention contact type
Interventions per month (for each strategy, months 1 -6)
Contact type by targeted antibiotic

- Vancomycin yes/no
- Piperacillin-Tazo yes/no
- Carbapenem yes/no

Targeted Antibiotic Vancomycin

- Targeted antibiotic Vancomycin (yes/no)
- If Yes, then
  - Vancomycin Recommendation
  - De-escalation (yes/no)
  - Continue Therapy (yes/no)
  - Changed dose (yes/no)
  - Other
  - List of other, specifies
  - Duration (continuous)

Targeted Antibiotic Piperacillin-Tazo

- Targeted antibiotic Piperacillin-Tazo (yes/no)
- If yes, then
  - Piperacillin-Tazo Recommendation
  - De-escalation (yes/no)
  - Continue Therapy (yes/no)
  - IV to PO conversion
  - Changed dose (yes/no)
  - Other
    - List of other, specifies
  - Duration (continuous)

Targeted Antibiotic Carbapenem

- Targeted antibiotic Carbapenem (yes/no)
- If yes, then
    - Carbapenem Recommendation
    - De-escalation (yes/no)
    - Continue Therapy (yes/no)
    - IV to PO conversion
    - Changed dose (yes/no)
    - Other
    - List of other, specifies
    - Duration (continuous)

Indication (all are yes/no)

- High risk of MDR infection
    - If yes, then (% among people at high risk of MDR, or % of total?)
    - History of MDR infection
    - Hospitalization in last 90 days
    - LTC facility in last year
    - Antibiotic or chemo in last 30 days
    - Hemodialysis (current)?
    - Other
    - List of other, specifies
    - Empiric need
    - Sepsis
    - Pneumonia
    - UTI
    - Meningitis
    - Surgical Site Infection
    - SSTI
    - Bacteremia
    - Other
    - List of other specifies
- Number of indications per intervention (not per person, as a given person could have more than one intervention over the course of a year

Signs and symptoms

- Fever
- Leukocytosis
- Hypotension
- Cough or Sputum production
- Other signs or symptoms

- List of other/specifies
- Number of signs and symptoms per intervention

Culture data

- Culture data available for review (yes/no)
- If yes, was the culture data reviewed
- If yes, did it help inform the recommendation?

Intervention outcome

- Was the recommendation followed?
- Was there an ID consult after the intervention?
- ICU admission

Ultimate Infection diagnosis related to recommendation

- Pneumonia
- UTI
- Meningitis
- Surgicial site infection
- SSTI
- Colitis
- Bacteremia
- Sepsis with unknown source
- No infection
- Other
- List of other, specify

10. Days Of Therapy

Purpose: To address Secondary objective 2, to compare DOT for antibiotics of interest between each strategy.

- Days of therapy (DOT) for targeted and alternative antibiotics
  - Per the protocol, DOT will be measured as the number of days a patient receives each targeted and alternative antibiotic during the hospital admission

The three targeted antibiotics are:

- Anti-pseudomonal carbapenem of choice at the study hospital
- Vancomycin
- Piperacillin-tazobactam.

The alternative antimicrobials of interest are:

- Fluoroquinolones
- Cephalosporins
- anti-MRSA systemic antimicrobials (e.g., nafcillin, daptomycin, linezolid, ceftaroline, clindamycin, and TMP-SMX).

Among admissions with an included antibiotic, by hospital

- Table 1
    - Year prior to study
    - Period 1 Admission with Included antibiotic, no intervention
    - Period 1 Admission with stewardship intervention
    - Period 2 admission with Included antibiotic, no intervention
    - Period 2 admission with stewardship intervention
- Table 2
    - Period 1 (all admissions with an antibiotic of interest)
    - Period 2 (all admissions with an antibiotic of interest)
- Table 3
    - For 3 contrasts (PAR vs Historical year; Res/PreA vs historical Year; PAR vs Res/PreA)
    - Difference in proportion used drug with 95%CI
    - Difference in Mean DOT with 95% CI

For each antibiotic,

- proportion of admissions where the antibiotic was received
- DOT for the antibiotic
    - Admissions where the antibiotic was not received will have a DOT of 0.

- Modeling (1 model for each of the antibiotics of interest)
    - The purpose of the model is to look at the effect of the intervention on DOT for each antibiotic of interest
    - Dependent variable = DOT for each antibiotic
    - Adjusting for hospital, study period, AS strategy, calendar time, demographic variables
    - The interactions of study period * strategy, hospital * study period, hospital * strategy, will be explored.

11. Prescriber and Pharmacist perceptions of the two stewardship strategies (Secondary objective 4)

- Summary tables of # respondents per hospital/period
- Summary of each survey question

12. Resource utilization (cost of antibiotics)

Purpose: to address secondary objective 1, to estimate and compare the resource utilization of each strategy where resource utilization includes cost of antibiotics

To be addressed following discussion of implementation of strategies given drug shortages and pending antibiotics cost information.

13. Patient specific outcomes between each strategy

Purpose: to address secondary objective 3, to compare patient-specific outcomes between each strategy, including but not limited to the following:

a. Total hospital days
b. Intensive care unit admission

Each of Total hospital days, death, and ICU admission will be summarized by hospital in 3 tables: Among admissions with an included antibiotic

- Table 1
    - Year prior to study
    - Period 1 Admission with Included antibiotic, no intervention
    - Period 1 Admission with stewardship intervention
    - Period 2 admission with Included antibiotic, no intervention
    - Period 2 admission with stewardship intervention
- Table 2
    - Period 1 (all admissions with an antibiotic of interest)
    - Period 2 (all admissions with an antibiotic of interest)
- Table 3
    - For 3 contrasts (PAR vs Historical year; Res/PreA vs historical Year; PAR vs Res/PreA)
    - Difference in proportion of ICU admissions with 95%CI
    - Difference in Mean total hospital days with 95% CI
